# Loss of heterozygosity: what is it good for?

**DOI:** 10.1186/s12920-015-0123-z

**Published:** 2015-08-01

**Authors:** Georgina L. Ryland, Maria A. Doyle, David Goode, Samantha E. Boyle, David Y.H. Choong, Simone M. Rowley, Jason Li, Australian Ovarian Cancer Study Group, David DL Bowtell, Richard W. Tothill, Ian G. Campbell, Kylie L. Gorringe

**Affiliations:** Cancer Genetics Laboratory, Peter MacCallum Cancer Centre, East Melbourne, Victoria Australia; Centre for Cancer Research, Monash Institute of Medical Research, Monash University, Clayton, Victoria Australia; Bioinformatics Core Facility, Peter MacCallum Cancer Centre, East Melbourne, Victoria Australia; Bioinformatics and Cancer Genomics Laboratory, Peter MacCallum Cancer Centre, East Melbourne, Victoria Australia; Sir Peter MacCallum Department of Oncology, University of Melbourne, Parkville, Victoria Australia; Peter MacCallum Cancer Centre, East Melbourne, Victoria Australia; Cancer Genomics and Genetics Laboratory, Peter MacCallum Cancer Centre, East Melbourne, Victoria Australia; Molecular Genomics Core Facility, Peter MacCallum Cancer Centre, East Melbourne, Victoria Australia; Department of Pathology, University of Melbourne, Parkville, Victoria Australia

**Keywords:** Tumour suppressor gene, SNP, Ovarian cancer, Mutation, Haploinsufficiency

## Abstract

**Background:**

Loss of heterozygosity (LOH) is a common genetic event in cancer development, and is known to be involved in the somatic loss of wild-type alleles in many inherited cancer syndromes. The wider involvement of LOH in cancer is assumed to relate to unmasking a somatically mutated tumour suppressor gene through loss of the wild type allele.

**Methods:**

We analysed 86 ovarian carcinomas for mutations in 980 genes selected on the basis of their location in common regions of LOH.

**Results:**

We identified 36 significantly mutated genes, but these could only partly account for the quanta of LOH in the samples. Using our own and TCGA data we then evaluated five possible models to explain the selection for non-random accumulation of LOH in ovarian cancer genomes: 1. Classic two-hit hypothesis: high frequency biallelic genetic inactivation of tumour suppressor genes. 2. Epigenetic two-hit hypothesis: biallelic inactivation through methylation and LOH. 3. Multiple alternate-gene biallelic inactivation: low frequency gene disruption. 4. Haplo-insufficiency: Single copy gene disruption. 5. Modified two-hit hypothesis: reduction to homozygosity of low penetrance germline predisposition alleles. We determined that while high-frequency biallelic gene inactivation under model 1 is rare, regions of LOH (particularly copy-number neutral LOH) are enriched for deleterious mutations and increased promoter methylation, while copy-number loss LOH regions are likely to contain under-expressed genes suggestive of haploinsufficiency. Reduction to homozygosity of cancer predisposition SNPs may also play a minor role.

**Conclusion:**

It is likely that selection for regions of LOH depends on its effect on multiple genes. Selection for copy number neutral LOH may better fit the classic two-hit model whereas selection for copy number loss may be attributed to its effect on multi-gene haploinsufficiency. LOH mapping alone is unlikely to be successful in identifying novel tumour suppressor genes; a combined approach may be more effective.

**Electronic supplementary material:**

The online version of this article (doi:10.1186/s12920-015-0123-z) contains supplementary material, which is available to authorized users.

## Background

Cancer cells undergo multiple genetic and epigenetic hits in the development of tumorigenic phenotypes, including somatic point mutations, increases in copy number, gene deletions, gene rearrangements, translocations and promoter hypermethylation [1]. These random events are selected for due to their effect on oncogenes, where the aberration activates the gene to promote tumorigenesis (e.g. *KRAS*, *MYC*), and on tumour suppressor genes (TSG), where the genetic or epigenetic aberrations is inactivating (e.g. *TP53*, *PTEN*), since the normal function of these genes is to restrict tumorigenic potential.

Loss of heterozygosity (LOH) is a common genetic event in many cancer types, so-called because of the early observations of a change in polymorphic markers from a heterozygous state in the germline to an apparently homozygous state in the tumour DNA [2]. LOH is a general term that encompasses both LOH with copy number losses (CNL-LOH) and copy number neutral LOH (CNN-LOH). In CNL-LOH all or part of a chromosome is deleted. CNN-LOH originates either through a homologous recombination event (“gene conversion”), or because the retained chromosome was duplicated either before or after the LOH event. LOH is strongly associated with loss of the wild-type allele in individuals with an inherited cancer predisposition syndrome and carry a germline mutation in genes such as *RB1* in retinoblastoma or *BRCA1* in breast and ovarian cancer [2, 3]. This “second hit” hypothesis was initially proposed by Knudson based on his observations of the incidence of familial retinoblastoma [4] and has been widely accepted as a mechanism for the complete inactivation of tumour suppressor genes, both in a germline context and the sporadic cancer context where the first hit is a somatic event, such as mutation of *TP53*. As a consequence, mapping of common regions of minimal LOH has historically been a popular strategy to pursue the identification of novel TSGs without the need for segregation data from large cancer families. However, such analyses have been generally been unsuccessful leading to speculation that the approach is technically and conceptually flawed [5], and even to whether there is any selective advantage to LOH events. Nonetheless, we previously used SNP mapping arrays to analyse LOH in ovarian carcinomas of diverse histological subtypes, with the rationale that the newer methodology would at least overcome some of the previous technical issues with LOH analyses [6]. We mapped a number of minimal regions of LOH containing tumour suppressor gene candidates, including regions of homozygous deletion encompassing genes such as *MAP2K4* [7]. Advances in massively parallel sequencing has enabled the current study where we report targeted sequencing of 980 candidate tumour suppressor genes in 86 ovarian carcinomas, most of which have matched SNP array data enabling the assessment of the importance of LOH in the selection for somatic mutations in ovarian cancer. We evaluated a number of different histological subtypes, since these have different etiologies and causative genes.

## Methods

### Ethics statement

Accrual and use of patient material for this study was approved by the following Human Research Ethics Committees: Peter MacCallum Cancer Centre Human Research Ethics Committee, Southampton Hospital Human Research Ethics Committee, University of Melbourne Human Research Ethics Committee, Queensland Institute of Medical Research Human Research Ethics Committee, Westmead Hospital Human Research Ethics Committee. All individuals gave written informed consent for the use of their tissue in research. This project was approved by the Peter MacCallum Cancer Centre Human Research Ethics Committee (Approval # 09/29).

### Ovarian tumour cohort

A tumour cohort (n = 86) comprising a variety of histological subtypes including serous (n = 45), endometrioid (n = 28), mucinous (n = 7) and clear cell (n = 6) were obtained through the Australia Ovarian Cancer Study, the Peter MacCallum Cancer Centre Tissue Bank, or from patients presenting to hospitals in the south of England [8]. The majority of tumour DNA samples were needle microdissected to ensure greater than 70 % cancer epithelial cell component; other samples were processed from tissue where the reference haematoxylin and eosin stained section showed >70 % tumour epithelial cells. Matching peripheral blood samples were also collected from patients at time of tumour collection and used as a source of germline DNA for somatic mutation detection. Details of the cohort are listed in Additional file 1: Table S1.

### Library preparation, target enrichment and sequencing

Library preparation was performed as previously described [9] following the Illumina genomic DNA library preparation protocol (Illumina, San Diego, CA) using an input of 200 ng of tumour or matched normal lymphocyte DNA. Seven custom multiplexing adapters compatible with Illumina single-end sequencing were used and indexed DNA samples were pooled equally prior to PCR enrichment. A boutique exon capture (SureSelect, Agilent Technologies, Santa Clara, CA) was used to enrich for coding exons of candidate tumour suppressor genes (n = 980, Additional file 1: Tables S2 and S3) and known cancer genes (*TP53*, *BRCA1*, *BRCA2*) according to the recommended protocol. Capture probes were designed using default parameters in eArray (Agilent Technologies).

Sequencing of target-enriched DNA libraries were performed using an Illumina GAIIx, generating 75 bp single-end sequence reads. Image analysis and base calling was performed using the Genome Analyser Pipeline v1.5-1.7. Sequence reads were aligned to the human reference genome (GRCh37/hg19 assembly) using BWA [10] and any remaining unmapped reads aligned with Novoalign [11]. The mean coverage for bases within target regions was 70-fold and 92 % had at least 10-fold coverage. This was followed by local realignment with GATK [12]. Point mutations and insertions/deletions (indels) were identified using GATK and Dindel [13] respectively, and annotated according to Ensembl release 56. Sequence variants were called as somatic alterations only when (*i*) the variant was not called in the matched normal sample or identified as a germline alteration in another tumour/normal pair (*ii*) the variant was not seen in > =2 independent reads in the matched normal sample following manual inspection of sequence reads using the Integrated Genomics Viewer [14] (*iii*) the variant was identified in bi-directional sequence reads.

A selection of variants that met the above criteria for a somatic mutation (n = 202) were subjected to validation by conventional PCR amplification and bidirectional capillary electrophoresis on the ABI3130 Genetic Analyser using BigDye Terminator v3.1 sequencing chemistry (Applied Biosystems, Foster City, CA).

### SNP arrays and loss of heterozygosity

Affymetrix SNP Mapping array data was obtained for the 86 sequenced cases, 54 by SNP6 arrays (GSE19539, [15]), 26 by 500 K arrays (previously published in [6]), and six previously unreported low-grade endometrioid cases. Affymetrix SNP6 CEL files, HM27 methylation array data (level 3), Agilent expression array data (level 3) and somatic mutation data from 266 tumors generated by The Cancer Genome Atlas (TCGA) were downloaded from the TCGA Data Portal. LOH was detected as described previously in Partek Genomics Suite (Partek, St Louis, MO), using allele-specific copy number that compared the tumour genotype to the matching normal genotype, and evaluated the copy number at heterozygous alleles [6]. The “min” allele had to have a value of <0.5 copies to be called LOH, thus excluding regions of allelic imbalance where at least one copy of both alleles was present. The results published here are in whole or part based upon data generated by The Cancer Genome Atlas pilot project established by the NCI and NHGRI. Information about TCGA and the investigators and institutions who constitute the TCGA research network can be found at http://cancergenome.nih.gov.

## Results and discussion

### A candidate TSG screen in ovarian cancer – selection of genes from LOH regions

Candidate ovarian tumour suppressor genes (n = 980) were selected for analysis on the basis of their location in frequent regions of LOH or deletion (Additional file 1: Tables S2 and S3) from our previously published SNP array analysis of 122 primary ovarian carcinomas of various histologies [6]. The regions, located on 20 different chromosome arms, met the following three criteria. Firstly, minimal overlapping regions of LOH were included if they were detected in greater than 35 % of all ovarian carcinomas analysed, or secondly, in >35 % of subtype specific minimal overlapping regions of LOH (4 of 9 clear cell carcinomas, 5 of 12 low-grade endometrioid carcinomas (grades 1 and 2), 6 of 16 mucinous carcinomas and 23 of 64 high-grade serous/endometrioid carcinomas (grades 2 and 3 for the serous subtype, grade 3 for endometrioid carcinomas)). Finally, all homozygous deletions within frequent regions of LOH along with the overlapping portion of all recurrent homozygous deletions were included. This gene list included genes with well established roles in cancer such as *CDKN2A* and *PTEN*, but for the purposes of this analysis they were included in the “candidate” LOH genes. In addition, the known ovarian cancer genes *TP53*, *BRCA1* and *BRCA2* were included despite lying outside the minimal regions of LOH.

### A candidate TSG screen in ovarian cancer – correlation of mutations with LOH

A targeted mutation screen was conducted on the 86 ovarian cancer cases including high-grade serous and endometrioid, low-grade endometrioid, clear cell and mucinous subtypes. Somatic coding mutations were detected in both candidate (561 variants in 366 genes, Additional file 2: Table S4) and known cancer genes (58 *TP53* mutations in 56 cases and two mutations in *BRCA1*). Eighty-nine genes had two or more non-synonymous mutations. The classic two-hit hypothesis would predict that driver genes have homozygous, deleterious mutations in samples with LOH. With respect to deleterious mutation status this was certainly true for *TP53* and *BRCA1* where a high proportion of somatic mutations were truncating (25/58 and 2/2, respectively) compared to an overall truncating mutation frequency of 13 % (72/561). In addition, among the 53 cases with *TP53* or *BRCA1* somatic mutations where SNP data was available, 50 (94 %) showed LOH of the wild-type allele. This was is sharp contrast with the other candidate genes, where only 181/520 showed LOH of the wild-type allele (35 %); in particular, there was no significant difference in LOH of the wild-type allele between non-synonymous mutations (134/381 with LOH, 35.2 %) and synonymous mutations (47/139 with LOH, 33.8 %). The overall frequency of non-synonymous compared to synonymous mutations was 73 % (411/561) for the candidate TSGs, but 100 % of mutations in known cancer genes were non-synonymous (60/60). This difference in ratio suggests that the majority of mutations in candidate TSGs from LOH regions are likely to be passenger events, since this rate might be expected without any strong positive selection [16]. The lack of difference in LOH between synonymous and non-synonymous also implies that there is limited selection for homozygosity for the majority of gene mutations.

### Significance analysis of recurrently mutated gene candidates

Within the list of mutated genes, we applied a number of filters to assess whether any genes could function as tumor suppressors under either a one-hit or two-hit mechanism. Firstly, significantly mutated genes were identified using the MuSiC algorithm [17], which determines the significance of the observed mutation rate of each gene based on the background mutation rate in the sample cohort. Three known ovarian cancer genes (*TP53*, *PTEN* and *CDKN2A*) were identified by all three tests (convolution, likelihood ratio and Fisher’s combined p-value tests) with a false discovery rate (FDR) of less than 0.10. At this FDR the genes *DNAH9*, *LINGO1*, *MEF2C*, *SAMD11*, *STARD5*, *ZNRF4* and *ZNF287* were also identified, although each was supported only by the likelihood ratio test.

Secondly, the 125 genes with recurrent mutations were assessed for the proportion of cases with biallelic mutation, including by homozygous deletion from SNP array data. For genes with mutations in three or more cases, the proportion of biallelic mutations was greater than 80 % of mutated samples for eight genes (*AL355987.1*, *CASK*, *CDKN2A*, *MAP2K4*, *NF1*, *PTEN*, *RB1* and *TP53*) and between 60**-**80 % for seven genes (*FANCA*, *GRAMD4*, *GPR98*, *IL16*, *MYOCD*, *SYNE1* and *TEX15*). Biallelic mutations were detected in 2/2 mutated samples in an additional nine genes (*SKG223*, *APOOL*, *BRCA1*, *CDH8*, *DACH2*, *EPHX2*, *FARP1*, *PNMA3* and *RAI1*).

Finally, genes recurrently targeted by inactivating mutations were identified. Mutations with overtly deleterious consequences were considered for this analysis, including nonsense and essential splice site mutations, frameshift indels and gene deletions. Although missense amino acid changes and in-frame indels can also negatively impact gene function, interpreting these mutations in the absence of functional validation is challenging. Sixteen genes were identified where more than half of their mutations would be considered clearly deleterious, including seven known ovarian cancer genes (*PTEN*, *CDKN2A*, *MAP2K4*, *PIK3R1*, *RB1*, *FANCA* and *BRCA1*). These three analyses identified 36 genes as possible tumour suppressors (Table 1), and it was notable that seven well characterised tumour suppressors were identified by at least two of the three methods (*BRCA1*, *TP53*, *RB1*, *PTEN*, *CDKN2A*, *FANCA*, and *MAP2K4*), although others were only identified by one method (*NF1*, *PIK3R1*). In contrast, 22 of the 27 (81 %) novel/less well characterized genes were identified by only 1 method, indicating that regions of LOH are not strongly enriching for novel genes with classic tumor suppressor gene characteristics.

**Table 1:** Selected mutated genes in candidate TSG screen

Gene	Location	Description	Recessive TSG^a^	Predominant subtypes	TCGA mutated^b^	Detected by
*ANKRD32*	5q15	ankyrin repeat domain 32		HG S/E + LG E + CC	9	Deleterious
*APOOL*	Xq21.1	apolipoprotein O-like		HG S/E	0	Biallelic
*BRCA1*	17q21.31	breast cancer 1, early onset	Y	HG S/E	12	Biallelic, Deleterious
*C9ORF172*	9q34.3	chromosome 9 open reading frame 172		HG S/E	5	Biallelic
*CACNA1B*	9q34	calcium channel, voltage-dependent, N type, alpha 1B subunit		LG E + CC	7	Deleterious
*CASK*	Xp11.4	calcium/calmodulin-dependent serine protein kinase		HG S/E	2	Biallelic
*CDH8*	16q22.1	cadherin 8, type 2		HG S/E	9	Biallelic
*CDKN2A*	9p21	cyclin-dependent kinase inhibitor 2A	Y	Muc	8	MuSiC, Biallelic, Deleterious
*CYLC1*	Xq21.1	cylicin, basic protein of sperm head cytoskeleton 1		HG S/E	4	Deleterious
*DACH2*	Xq21.3	dachshund homolog 2 (Drosophila)		HG S/E	3	Biallelic
*DNAH9*	17p12	dynein, axonemal, heavy chain 9		LG E	16	MuSiC
*EPHX2*	8p21	epoxide hydrolase 2, cytoplasmic		HG S/E	12	Biallelic
*FANCA*	16q24.3	Fanconi anemia, complementation group A	Y	HG S/E	12	Biallelic, Deleterious
*FARP1*	13q32.2	FERM, RhoGEF (ARHGEF) and pleckstrin domain protein 1		HG S/E	3	Biallelic
*GPR98*	5q13	G protein-coupled receptor 98		HG S/E	17	Biallelic
*GRAMD4*	22q13.31	GRAM domain containing 4		HG S/E	18	Biallelic, Deleterious
*IL16*	15q26.3	interleukin 16		HG S/E	4	Biallelic, Deleterious
*LINGO1*	15q24.3	leucine rich repeat and Ig domain containing 1		HG S/E	4	MuSiC
*MAP2K4*	17p12	mitogen-activated protein kinase kinase 4	Y	HG S/E	12	Biallelic, Deleterious
*MEF2C*	5q14.3	myocyte enhancer factor 2C		HG S/E	8	MuSiC
*MYOCD*	17p11.2	myocardin		HG S/E	7	Biallelic, Deleterious
*NF1*	17q11.2	neurofibromin 1	Y	HG S/E	37	Biallelic
*PIK3R1*	5q13.1	phosphoinositide-3-kinase, regulatory subunit 1 (alpha)	Y	LG E	6	Deleterious
*PNMA3*	Xq28	paraneoplastic Ma antigen 3		HG S/E	2	Biallelic
*PTEN*	10q23	phosphatase and tensin homolog	Y	LG E	25	MuSiC, Biallelic, Deleterious
*RAI1*	17p11.2	retinoic acid induced 1		HG S/E	4	Biallelic
*RB1*	13q14.2	retinoblastoma 1	Y	HG S/E	32	Biallelic, Deleterious
*RPS6KA6*	Xq21.1	ribosomal protein S6 kinase, 90 kDa, polypeptide 6		HG S/E + LG E	0	Deleterious
*SAMD11*	1p36.33	sterile alpha motif domain containing 11		HG S/E	7	MuSiC, Deleterious
*SKG223*	8p23.1	Sugen kinase 223		HG S/E + Muc	7	Biallelic
*STARD5*	15q26	StAR-related lipid transfer (START) domain containing 5		HG S/E	1	MuSiC
*SYNE1*	6q25	spectrin repeat containing, nuclear envelope 1		HG S/E	14	Biallelic
*TEX15*	8p22	testis expressed 15		HG S/E	13	Biallelic
*TP53*	17p13.1	tumor protein p53	Y	HG S/E	302	MuSiC, Biallelic
*ZNF287*	17p11.2	zinc finger protein 287		HG S/E	4	MuSiC, Deleterious
*ZNRF4*	19p13.3	zinc and ring finger 4		HG S/E	1	MuSiC

LG E, low-grade endometrioid; HG S/E, high-grade serous/endometrioid; Muc, mucinous; CC, clear cell

^a^Known recessive tumour suppressor gene according to the Cancer Gene Census [32]

^b^Number of high-grade serous TCGA samples with somatic point mutations and indels including large homozygous deletions. Mutation data for 316 TCGA samples [33] was accessed through the cBio Cancer Genomic Portal [34]

### Loss of heterozygosity – what is it good for?

**Fig. 1:**
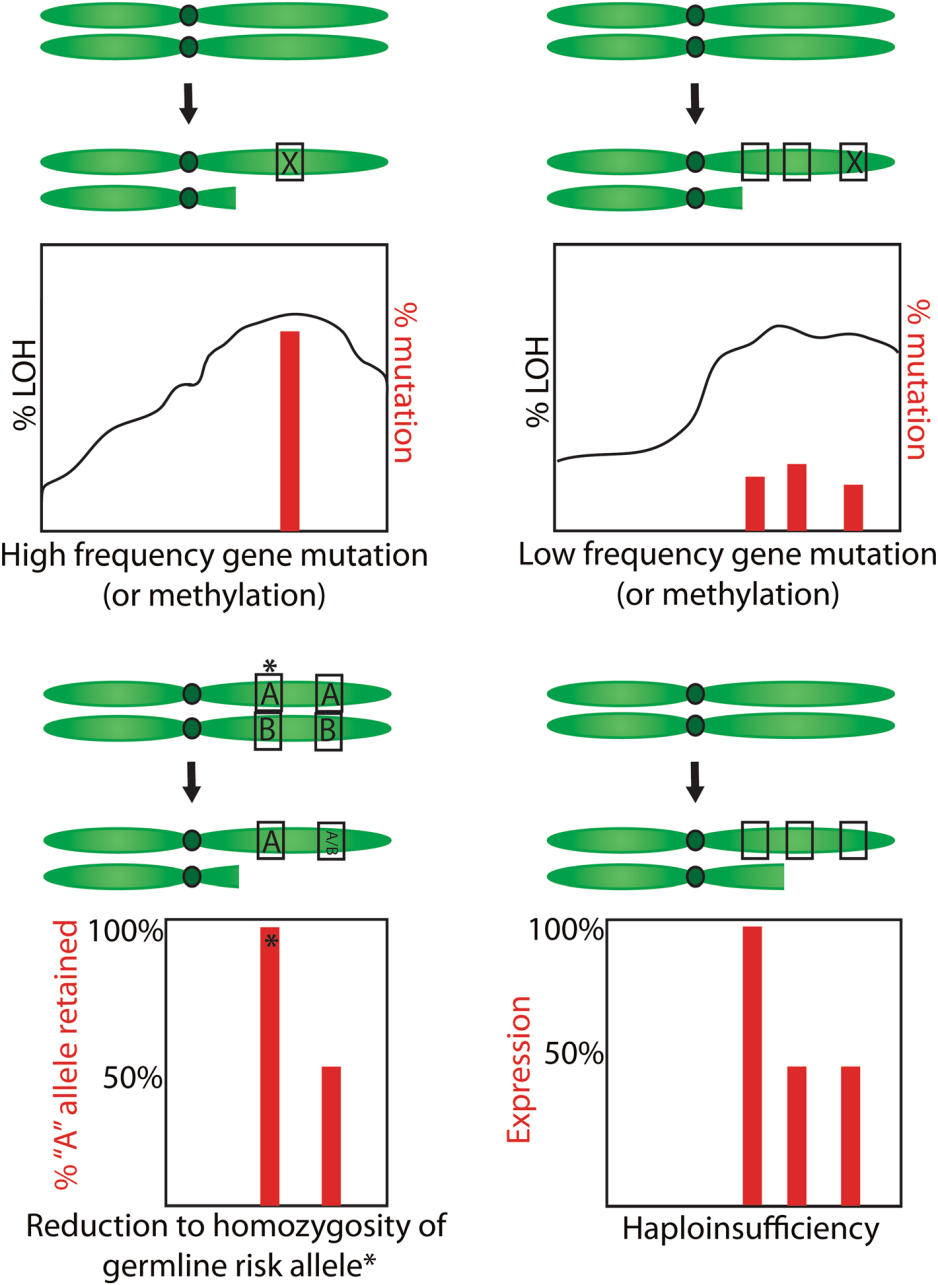
Models of LOH. Boxes = genes; “X” = inactivating mutation; A, B = alternative alleles of a single nucleotide polymorphism. In the top panels, the black line on the graph represents the overall frequency of LOH observed in tumour samples across the chromosome, while the red bars are the frequency of mutation in a particular gene. Thus, for the classic two-hit model, the frequency of mutation is similar to the frequency of LOH, while in the low frequency model, the frequency of LOH is higher than the mutation rate, because each sample is mutated in a different gene. In the bar graphs below, at left, the red bars represent the frequency of the A allele that is retained in samples with LOH at the locus; thus, the risk locus (*) has a higher proportion of the risk allele (A) retained after LOH compared to a non-risk locus, where the A and B alleles are equally retained. At right, the graphs represents the average reduction in expression of a gene in samples with LOH, compared to samples without LOH; genes in LOH regions show a reduction in expression

From the data above it appears that we did not identify dominant, very frequently mutated novel genes where selection for a classic two-hit tumour suppressor gene was apparent. So what, if anything, is the LOH for? We considered five possibilities (Fig. 1) and assessed each in turn.

1. Classic two-hit hypothesis: high frequency biallelic genetic inactivation of TSG
2. Epigenetic two-hit hypothesis: biallelic inactivation through methylation and LOH
3. Multiple alternate gene biallelic inactivation: low frequency gene disruption
4. Haplo-insufficiency: Single copy gene disruption
5. Modified two-hit hypothesis: reduction to homozygosity of predisposition alleles

1. Classic two-hit hypothesis: high frequency biallelic genetic inactivation of TSG

This mechanism is demonstrably true for many known tumour suppressor genes, with *TP53* being a clear example of a gene functioning as a classical TSG in ovarian cancer [18, 19]. However, from our data and large published studies such as TCGA, it is clear that novel genes with a high frequency of biallelic mutations are exceedingly rare and can not explain the bulk of the observed LOH. For example, 8p undergoes LOH in >40 % of ovarian carcinomas, but no gene in this region is mutated at frequency higher than 3 % in our or any other study, although homozygous deletion can target, for example, *CSMD1* in 11 % of cases [20]. It remains a possibility, however, that genes not represented on our targeted or exome sequencing platforms could still be the target of such LOH, for example long non-coding RNAs.

2. Epigenetic two-hit hypothesis: high frequency biallelic inactivation through methylation and LOH

**Fig. 2:**
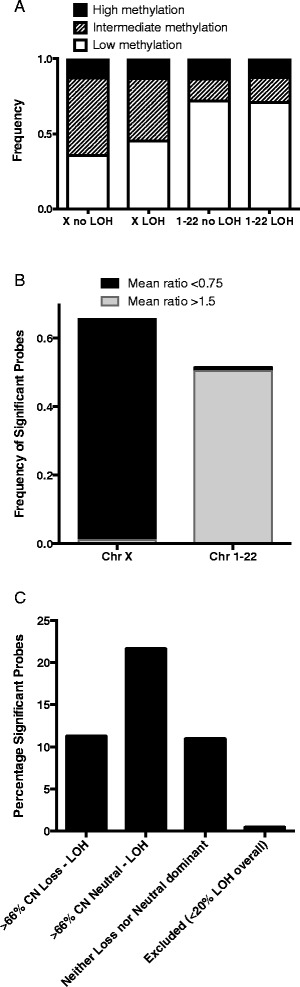
Methylation. **a** Frequency of probes on the HM27 methylation array that have high (value > 0.75), intermediate (0.25-0.75) and low (<0.25) methylation associated with LOH in a sample, comparing all autosomes (no difference between LOH and no LOH) and chromosome X (more low methylation probes in LOH regions). **b** Considering only probes that were significantly different between LOH and no LOH, the frequency of significant probes where the mean methylation ratio (LOH/no LOH) was increased (>1.5, higher in LOH) or decreased (<0.75, higher in no LOH). **c** Percentage of significant methylation probes that are located in regions where the majority (>2/3) of LOH is either copy number loss, neutral or neither. Only regions with at least 20 % LOH were included

Somatic gene mutation is not the only mechanism of biallelic inactivation. Some TSGs can be inactivated through a combination of LOH and promoter hypermethylation, for example *MLH1*. This methylation can be acquired somatically or may be a consequence of imprinting. We assessed this possibility using TCGA ovarian cancer methylation data. Globally, we observed that there was no enrichment for methylation in regions of LOH – in samples with LOH at a locus, on average 12.7 % of genes were strongly methylated (probe value of >0.75), whereas 13.65 % of genes were strongly methylated when there was no LOH, (Fig. 2a). CNL-LOH was less likely to have strongly methylated genes than CNN-LOH (12.3 % vs 12.9 %, p < 0.0001, Chi-squared test). However, when we analysed the X chromosome separately, we found that samples with any LOH were more likely to have low methylation levels (45.3 % of genes had a probe value of <0.25, compared to 35.5 % in samples without LOH, p < 0.0001 Chi-squared test).

Detection of methylation is challenging from both technical and biological perspectives. Tumour and cell type heterogeneity may influence the degree of methylation detected, so we also took an alternative approach where we used the methylation array data to test whether there were genes that were more strongly methylated in samples with LOH compared to samples without LOH. Using a multiple testing correction p-value threshold of 2.2x10^−6^, there were 1584/22374 (7 %) methylation probes that were significantly differentially methylated. Interestingly, 28 % of these significant probes were located on the X chromosome and indeed 51 % of all probes on the X chromosome were significantly differentially methylated, with lower average levels of methylation in samples with LOH compared to samples without LOH. On the autosomes, the outcome was reversed: 50.3 % of the statistically significant probes had a fold-change difference in mean methylation of >1.5, while only 1.1 % had a fold-change difference of <0.75 (Fig. 2b). Thus, for the X chromosome it appears there is selection for retaining the active copy, perhaps because loss of this copy would be cell lethal as an effective homozygous inactivation of the chromosome. In contrast, for the autosomes there appears to be selection for increased methylation by LOH.

We also evaluated whether there was any difference by the type of LOH. For those genes occurring in a region of LOH with at least 20 % frequency, we determined whether a probe was in a CNL-LOH enriched locus (>66 % of samples with LOH also had CN loss) or a CNN-LOH enriched locus (>66 % of samples with LOH were CNN). Of the CNL-enriched probes, 11.3 % were significantly differentially methylated, compared to 21.7 % of CNN-enriched probes (p < 0.0001, Chi-squared test, Fig. 2c). This data would support a model whereby differential methylation is more commonly selected for in regions of CNN-LOH than CNL-LOH.

3. Multiple alternate gene biallelic inactivation: low frequency gene disruption

Another possibility is that particular loci harbour multiple TSGs but individual tumours only require one to be inactivated and the gene targeted can differ from tumour to tumour. If this is the case then locating the TSGs by mapping overlapping regions of LOH would incorrectly flag the interval between two TSG as the likely location of the TSG – in effect then the peak LOH regions may not be the most likely places to find the targeted gene(s). To evaluate this possibility, we used TCGA data to see whether regions of LOH were enriched for somatic mutations on a sample-by-sample basis. Cases with both somatic exome and SNP array data were used (n = 266). There were 13,148 coding somatic mutations, of which 29.7 % were located within a region of LOH in the sample where it was observed. The average overlap of all the genes assayed with regions of LOH per sample was 35.5 %. Thus, somatic mutations are if anything under-represented in regions of LOH (Binomial test p < 0.0001). Given that most of these mutations are likely to be passengers, we evaluated whether this was true for non-synonymous or overtly deleterious mutations (nonsense, frameshift, essential splice site). For deleterious mutations, 38.2 % were in regions of LOH (p = 0.035, Binomial test), whereas only 25.2 % of the non-synonymous mutations were in regions of LOH, similar to the 22.2 % observed for synonymous mutations. The signal for deleterious mutations was substantially reduced if *TP53* was excluded (34.9 % of deleterious and 28.5 % of other non-synonymous mutations had LOH). Thus, regions of LOH are slightly enriched for deleterious mutations, but not for other non-synonymous or silent mutations.

**Fig. 3:**
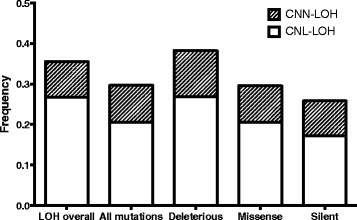
Mutation load. Frequency of mutations of various types in copy number neutral and copy number loss regions, compared to the overall frequency of LOH across the exome (“LOH overall”). Deleterious mutations are enriched in CNN-LOH; all other mutations types are less frequent in copy number loss regions

We then evaluated whether there was a difference in mutation frequency in CNN versus CNL regions of LOH on a case by case basis as above (Fig. 3). Excluding *TP53*, there were fewer mutations in regions of CNL-LOH than would have been expected based on the overall percentage of the exome affected (19.8 % of mutations were in CNL-LOH regions, whereas 26.8 % of the exome was affected by CNL-LOH, p < 0.0001, Binomial test). The difference was less striking when considering overtly deleterious mutations only (24.6 % vs 26.8 %, p = 0.09, Binomial test). For CNN-LOH, the overall difference was small (8.8 % of mutations vs 8.7 % of the exome affected by CNN-LOH, p = 0.7, Binomial test), however there were more deleterious mutations than expected (10.3 % vs 8.7 %, p = 0.05, Binomial test). When *TP53* was included, both total mutations (9.2 %) and deleterious mutations (11.4 %) in CNN-LOH regions were increased. Silent mutations were the most likely to be underrepresented in CNL-LOH regions (17.2 % vs 26.8 %). It is possible, therefore, that mutations are seen less often in CNL-LOH regions simply as a consequence of decreased DNA dosage. The enrichment of deleterious mutations in CNN-LOH regions, however, suggests the presence of positive selection for mutations in TSGs.

4. Haplo-insufficiency: Single copy gene disruption

We and others have shown that loss of a single gene copy can reduce gene expression [6, 21]. A recent study showed that regions of copy number loss are enriched for tumour-suppressor genes [22], but that each gene might have a limited effect on its own. Chromosome complementation studies, where all or part of a chromosome is introduced into cell lines with LOH of that chromosome *via* microcell-mediated monochromosome transfer, have frequently been able to show reduction in tumorigenicity of the cell line thus complemented [23, 24], but have only rarely been able to implicate a single gene responsible [25, 26]. Thus, haplo-insufficiency of multiple genes, each with a small effect, could contribute to the non-random pattern of LOH observed in ovarian cancer, especially for chromosomal regions that are weighted towards CNL-LOH such as 8p and X, rather than CNN-LOH, such as 17.

**Fig. 4:**
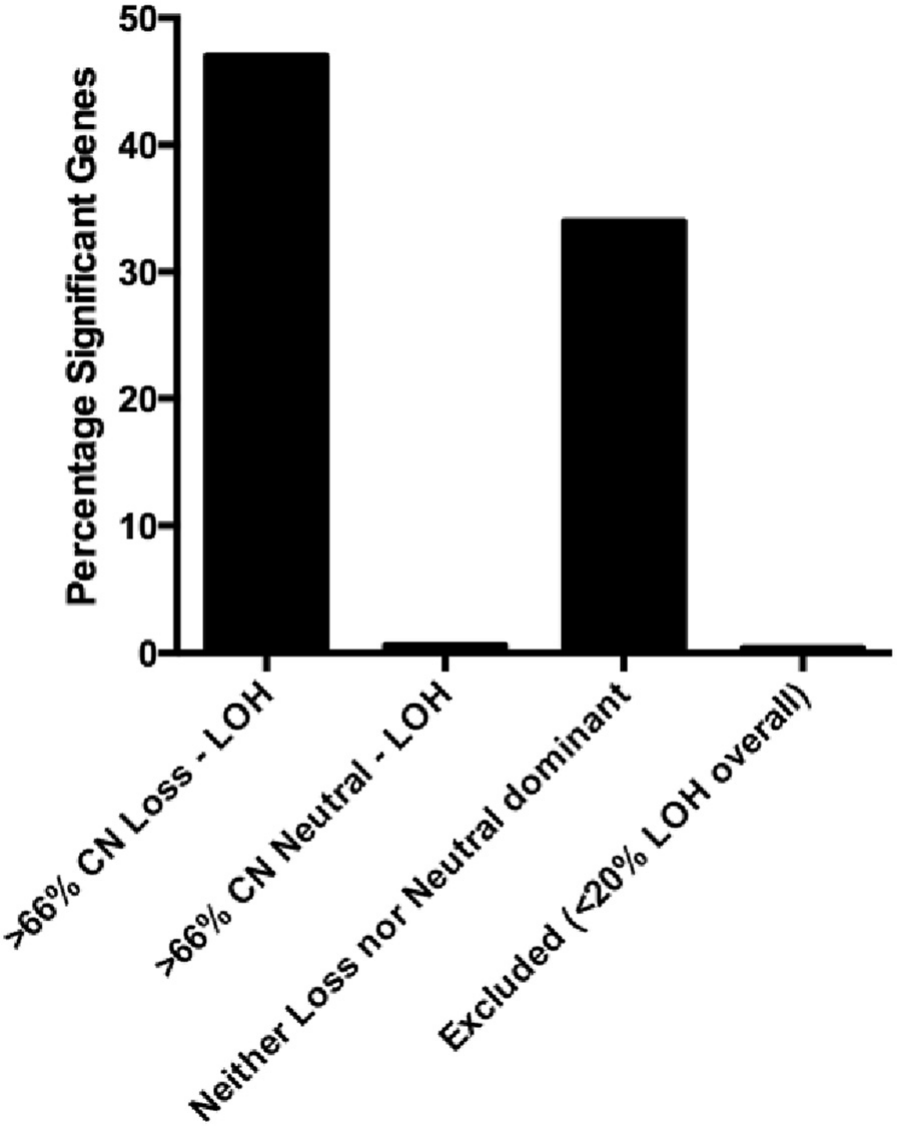
Expression. The percentage of significantly differentially expressed genes in regions where the majority (>2/3) of LOH is either copy number loss, neutral or neither. Only regions with at least 20 % LOH were included

We previously observed a correlation between the percentage of genes under-expressed and the percentage of cases with CNL-LOH, as opposed to CNN-LOH, in a region-wise comparison of LOH *vs.* no LOH [6]. In an analysis of TCGA data, we compared the expression of 10,925 genes between cases with and without LOH. 3,780 genes (34.6 %) were significantly differentially expressed (at a multiple testing p-value threshold of 4.56x10^−6^), and all significant genes were under-expressed in samples with LOH compared to samples without LOH. When comparing CNN-LOH to CNL-LOH in genes with at least 20 % frequency of LOH, only 1/163 genes (0.6 %) at CNN-LOH enriched loci were significantly differentially expressed, compared to 2701/5740 (47 %) CNL-LOH enriched genes (p < 0.0001, Chi-squared test, Fig. 4). This result supports the idea that chromosomal regions with CNL-LOH may contain genes where loss of a single copy results in reduced gene expression and a selective advantage to the cell. In contrast, chromosomal regions with little copy number loss may contain essential genes for which haplo-insufficiency is cell lethal.

5. Modified two-hit hypothesis: reduction to homozygosity of predisposition alleles

In familial cancer predisposition syndromes, it is common for the remaining wild-type allele to be lost by LOH, for example *BRCA1* pathogenic variants are usually reduced to homozygosity in breast and ovarian carcinomas [3, 27]. However, common low-penetrance risk alleles could also be targeted by LOH leading to an enhancement of their cancer-promoting role. We assessed this using nine SNP loci identified in the iCOGs study [28, 29] as predisposing to all ovarian cancer types or high-grade serous ovarian cancer. Two of these SNPs were present on the Affymetrix SNP 6.0 array, the remainder were represented by SNPs in linkage disequilibrium (r^2^ > 0.7, from HapMap [30]). Where possible, up to four linked SNPs were evaluated.

For each SNP, we assessed whether cases were heterozygous in their normal DNA, and what proportion of these with LOH of the region were homozygous for the risk allele in the tumour DNA using TCGA and our own data (n = 364). Interestingly, two SNPs at 10p12 linked to the risk allele rs1232180 were found to be significantly more likely to have lost the non-risk allele than the risk allele (Table 2). Some other SNPs linked to a risk allele also showed significantly non-random loss of the non-risk allele, but the data were not consistent across all SNPs examined at the locus (e.g. 17q21, 3q25 and 9p22). It is not clear whether these discrepancies could be due to technical variation in the SNP calling; alternative methods may be required to assess this possibility. The remainder of SNPs were not significant, however several are uncommon, limiting the power of the analysis. Thus, it is possible that some LOH may be selected for through the phenotypic effect of reduction to homozygosity of predisposition alleles.

**Table 2:** Ovarian cancer GWAS SNPs and LOH

SNP^a^	Risk Allele^b^	Locus	het^c^	hom	AA	BB	NC	N	CHI sq	Direction	% LOH^d^	N LOH	Affy SNP	Rsquared
rs1243180	NA	10p12												
rs1243188	minor/A	10p12	112	183	22	6	41	364	0.0025	yes	0.20	28	SNP_A-2024177	0.881
rs7098100	minor/B	10p12	132	181	1	9	41	364	0.0114	yes	0.07	10	SNP_A-8636193	0.781
rs757210	NA	17q12												
rs11658063	minor/B	17q12	23	148	44	44	105	364	1.0000	.	0.79	88	SNP_A-8714923	0.704
rs9303542	NA	17q21												
rs4451990	minor/B	17q21	20	197	49	61	37	364	0.2526	.	0.85	110	SNP_A-2282117	1
rs12944592	minor/B	17q21	24	198	54	60	28	364	0.5741	.	0.83	114	SNP_A-1836563	1
rs12452212	minor/A	17q21	19	196	74	53	22	364	0.0624	yes	0.87	127	SNP_A-2128564	1
rs9894812	minor/A	17q21	20	198	70	48	28	364	0.0428	yes	0.86	118	SNP_A-2209606	1
rs8170	NA	19p13												
rs34084277	minor/B	19p13	80	260	9	8	7	364	0.8084	.	0.18	17	SNP_A-1788674	1
rs2072590	NA	2q31												
rs711830	minor/B	2q31	77	160	8	14	105	364	0.2008	.	0.22	22	SNP_A-8652216	0.965
rs7651446	NA	3q25												
rs344008	minor/A	3q25	58	297	2	3	4	364	0.6547	.	0.08	5	SNP_A-8543714	0.85
rs2292336	minor/B	3q25	48	299	2	5	10	364	0.2568	.	0.13	7	SNP_A-8587822	0.85
rs17380639	minor/A	3q25	28	320	11	3	2	364	0.0325	yes	0.33	14	SNP_A-2078455	0.85
rs11782652	minor/B	8q21	38	291	8	7	20	364	0.7963	.	0.28	15	SNP_A-8702651	.
rs10088218	major/A	8q24	47	280	11	11	15	364	1.0000	.	0.32	22	SNP_A-1801410	.
rs1516974	major/A	8q24	45	291	4	15	9	364	0.0116	no	0.30	19	SNP_A-2088878	1
rs3814113	NA	9p22												
rs7032221	major/B	9p22	123	201	6	11	23	364	0.2253	yes	0.12	17	SNP_A-8603886	1
rs10738467	major/B	9p22	111	206	5	11	31	364	0.1336	yes	0.13	16	SNP_A-8328297	0.892
rs10962668	major/B	9p22	103	177	7	22	55	364	0.0053	yes	0.22	29	SNP_A-4198891	0.794

^a^ SNPs in bold are those named in the GWAS iCOG publication [29] All others are linked as indicated by the R-squared value of >0.7. If the minor allele is the risk allele named, it is assumed that this will also be the case for the linked SNP

^b^ Minor = risk allele is the less frequent allele in the population. A, B = risk allele corresponds to the “A” or “B” allele respectively in the Affymetrix array nomenclature. NA = not on Affymetrix SNP6 array

^c^ Het = Number of cases where germline and tumour are heterozygous, hom = cases where germline is heterozygous, AA, BB = germline is heterozygous, tumour is homozygous for A or B respectively, NC = no call in either tumour or germline. N = total number

^d^ % LOH is the number of individuals with loss of one allele divided by the total number of heterozygous individuals as measured at that SNP, i.e. not the overall % of LOH that could be determined from all cases using a wider genetic window. This may therefore include regions of extreme allelic imbalance (e.g. likely for 8q24)

## Conclusion

The broader relevance of LOH in cancer has been debated for some time [5, 31] although many of the criticisms stemmed from technical issues that are being overcome by newer methodologies. Our initial assumption for this study was that we would detect high-frequency mutated genes in the minimal peak regions of LOH we had defined by LOH mapping using these newer methodologies; i.e. a classic two-hit model. However, the biology of LOH does not support this assumption and with large-scale tumour studies it is now possible to explore the many possibilities for the functional significance of this genetic event as summarised in Table 3. We suggest that the non-random patterns of LOH detected in cancer are a result of multiple different mechanisms operating to affect multiple genes, which may differ from tumour to tumour yet collectively play a role in the development of the tumorigenic phenotype. It is worth noting the differences in CNL-LOH versus CNN- LOH, with the latter appearing more relevant for selection of deleterious mutations and methylation, in contrast to global changes in gene expression. Identifying the specific driver genes targeted in a particular cancer remains a challenge given the multiple possible reasons for selection of an LOH event.

**Table 3:** Summary of LOH – what is it good for?

Hypothesis	Mechanism	Plausibility	Frequency	Impact	LOH type
Classic two-hit hypothesis	High frequency biallelic genetic inactivation of TSG via mutation and LOH or homozygous deletion	Strong	Rare	High	More CNN-LOH
Modified two-hit hypothesis	Reduction to homozygosity of predisposition alleles	Low	Rare	Low	Unknown
Epigenetic two-hit hypothesis	Biallelic inactivation through methylation and LOH	Moderate	Unknown	Moderate	More CNN-LOH
Haplo-insufficiency	Single copy gene disruption through copy number loss	Strong	Common	Moderate	CNL-LOH
Multi-gene biallelic inactivation	Low frequency gene disruption through all of the above mechanisms	Strong	Common	Unknown	Either

## Additional files

Additional file 1: Table S1. Cohort information, **Table S2.** Regions of LOH analysed by targeted sequencing, **Table S3.** List of the genes in regions of LOH analysed by targeted sequencing. (DOCX 135 kb) Additional file 2: Table S4. Excel file with all synonymous and non-synonymous mutations identified by targeted sequencing. (XLSX 141 kb)

## Abbreviations

AOCS: Australian Ovarian Cancer Study
LOH: Loss of heterozygosity
CNL-LOH: Copy number loss LOH
CNN-LOH: Copy number neutral LOH
SNP: Single nucleotide polymorphism
TCGA: The Cancer Genome Atlas
TSG: Tumour suppressor gene
