# Supplementary material for: Loss of heterozygosity: what is it good for?
Source: BMC Med Genomics. 2015 Aug 1;8:45. doi: 10.1186/s12920-015-0123-z (PMC4522148; doi:10.1186/s12920-015-0123-z)
Supplement: Additional file 1: Table S1. — Cohort information, Table S2. Regions of LOH analysed by targeted sequencing, Table S3. List of the genes in regions of LOH analysed by targeted sequencing. (DOCX 135 kb) [file 12920_2015_123_MOESM1_ESM.docx]

Additional file 1

Table S1. Ovarian sample cohort.

| Sample | Subtype | Grade | Stage | Patient age | LOH data source^a^ |
| --- | --- | --- | --- | --- | --- |
| IC349T | Clear cell | 2 | NK | 39 | SNP6.0 |
| IC549T | Clear cell | 3 | NK | 42 | SNP6.0 |
| P0511T | Clear cell | 3 | IIB | 69 | SNP6.0 |
| P1094T | Clear cell | NK | IA | 56 | SNP6.0 |
| P4881T | Clear cell | 2 | III | 34 | 500K |
| P5390T | Clear cell | 3 | III | 51 | SNP6.0 |
| IC050T | Mucinous | 2 | I | 81 | 500K |
| IC080T | Mucinous | 1 | IA | 71 | 500K |
| IC138T | Mucinous | 1 | IA | 47 | 500K |
| IC219T | Mucinous | 1 | IC | 63 | 500K |
| IC257T | Mucinous | 1 | IA | 60 | SNP6.0 |
| IC343T | Mucinous | 1 | NK | 53 | 500K |
| IC448T | Mucinous | 1 | IC | 55 | SNP6.0 |
| 6389T | Endometrioid | 1 | NK | NK | SNP6.0 |
| 2108T | Endometrioid | 1 | NK | NK | SNP6.0 |
| 4961T | Endometrioid | 1 | NK | NK | SNP6.0 |
| 11307T | Endometrioid | 1 | NK | NK | SNP6.0 |
| 1513T | Endometrioid | 1 | NK | NK | SNP6.0 |
| 3321T | Endometrioid | 1 | NK | NK | SNP6.0 |
| P1921T | Endometrioid | 1 | IA | 63 | SNP6.0 |
| P1977T | Endometrioid | 1 | IIC | 74 | SNP6.0 |
| IC128T | Endometrioid | 2 | IC | 56 | SNP6.0 |
| IC151T | Endometrioid | 2 | IIC | 56 | SNP6.0 |
| IC220T | Endometrioid | 2 | IIIC | 62 | SNP6.0 |
| IC359T | Endometrioid | 2 | IA | 57 | 500K |
| IC410T | Endometrioid | 2 | III | 51 | 500K |
| IC526T | Endometrioid | 2 | III | 74 | 500K |
| IC580T | Endometrioid | 2 | IC | 70 | SNP6.0 |
| P1768T | Endometrioid | 2 | IA | 51 | SNP6.0 |
| IC095T | Endometrioid | 3 | II | 66 | SNP6.0 |
| IC179T | Endometrioid | 3 | III | 62 | SNP6.0 |
| IC258T | Endometrioid | 3 | IIIC | 69 | SNP6.0 |
| IC286T | Endometrioid | 3 | IIIB | 71 | 500K |
| IC293T | Endometrioid | 3 | I | 59 | SNP6.0 |
| IC300T | Endometrioid | 3 | III | 68 | 500K |
| IC399T | Endometrioid | 3 | IV | 66 | SNP6.0 |
| IC434T | Endometrioid | 3 | IC | 76 | SNP6.0 |
| IC504T | Endometrioid | 3 | IC | 60 | SNP6.0 |
| IC533T | Endometrioid | 3 | IV | 87 | 500K |
| IC594T | Endometrioid | 3 | I | 47 | SNP6.0 |
| P0505T | Endometrioid | 3 | II | 59 | 500K |
| IC487T | Serous | 1 | III | 41 | SNP6.0 |
| IC026T | Serous | 2 | IIIC | 51 | SNP6.0 |
| IC135T | Serous | 2 | II | 66 | 500K |
| IC201T | Serous | 2 | IIIB | 80 | SNP6.0 |
| IC328T | Serous | 2 | NK | 66 | SNP6.0 |
| IC407T | Serous | 2 | III | 83 | 500K |
| IC493T | Serous | 2 | III | 61 | SNP6.0 |
| IC509T | Serous | 2 | III | 66 | 500K |
| IC519T | Serous | 2 | IC | 64 | 500K |
| P0706T | Serous | 2 | IIIC | 57 | SNP6.0 |
| P1246T | Serous | 2 | IIC | 69 | SNP6.0 |
| P1428T | Serous | 2 | IIA | NK | SNP6.0 |
| P1805T | Serous | 2 | IIIB | 64 | SNP6.0 |
| P2803T | Serous | 2 | IIIC | NK | SNP6.0 |
| P4075T | Serous | 2 | IIIC | 54 | SNP6.0 UP |
| P7820T | Serous | 2 | IIC | NK | SNP6.0 UP |
| IC022T | Serous | 3 | III | 65 | SNP6.0 |
| IC086T | Serous | 3 | IIIA | 84 | 500K |
| IC131T | Serous | 3 | III | 74 | SNP6.0 |
| IC141T | Serous | 3 | IIIC | 56 | 500K |
| IC277T | Serous | 3 | IIIC | 71 | 500K |
| IC315T | Serous | 3 | NK | 82 | SNP6.0 |
| IC318T | Serous | 3 | III | 70 | SNP6.0 |
| IC325T | Serous | 3 | NK | 64 | SNP6.0 |
| IC382T | Serous | 3 | III | 59 | SNP6.0 |
| IC406T | Serous | 3 | NK | 76 | 500K |
| IC413T | Serous | 3 | III | 36 | SNP6.0 |
| IC489T | Serous | 3 | III | 42 | SNP6.0 |
| IC551T | Serous | 3 | III | 55 | 500K |
| P0667T | Serous | 3 | IIB | 56 | 500K |
| P0756T | Serous | 3 | II | 66 | 500K |
| P0933T | Serous | 3 | IIC | 85 | SNP6.0 |
| P1049T | Serous | 3 | II | 50 | SNP6.0 |
| P1348T | Serous | 3 | II | 41 | SNP6.0 |
| P1555T | Serous | 3 | IIB | 52 | SNP6.0 |
| P2205T | Serous | 3 | I | 50 | SNP6.0 UP |
| P2712T | Serous | 3 | IA | NK | SNP6.0 UP |
| P4076T | Serous | 3 | IIIA | 56 | SNP6.0 UP |
| P4093T | Serous | 3 | IIIC | 60 | SNP6.0 UP |
| P4178T | Serous | 3 | III | NK | SNP6.0 |
| P4219T | Serous | 3 | IIIC | 44 | SNP6.0 |
| P4616T | Serous | 3 | IC | 52 | SNP6.0 |
| P4684T | Serous | 3 | IIIC | 73 | SNP6.0 |
| P5338T | Serous | 3 | IIIC | 40 | 500K |
| P5514T | Serous | 3 | IIIC | 69 | 500K |

NK, not known.

^a^Refers to Affymetrix SNP6.0 or 500K microarray data used to identify regions of LOH in each sample. UP = unpaired data; i.e. lacking matching normal SNP6.0 data and not used for LOH comparisons.

**Table S2. LOH regions for candidate TSG sequencing (hg19)**

| Chromosome | Start | End |
| --- | --- | --- |
| 1 | 10,002 | 1,018,704 |
| 1 | 1,746,695 | 3,504,073 |
| 1 | 5,597,414 | 8,066,914 |
| 1 | 20,502,362 | 20,895,797 |
| 1 | 22,499,210 | 22,522,239 |
| 4 | 92,625,771 | 92,661,406 |
| 4 | 94,208,316 | 94,337,840 |
| 4 | 149,883,034 | 149,936,795 |
| 4 | 151,203,823 | 151,469,688 |
| 4 | 152,785,930 | 152,792,729 |
| 4 | 156,137,788 | 156,216,750 |
| 4 | 161,877,010 | 161,884,592 |
| 4 | 187,737,007 | 191,029,082 |
| 5 | 59,394,244 | 59,414,243 |
| 5 | 62,483,935 | 62,508,902 |
| 5 | 67,512,156 | 67,528,190 |
| 5 | 67,560,866 | 67,589,410 |
| 5 | 74,414,340 | 74,513,263 |
| 5 | 75,915,245 | 76,340,244 |
| 5 | 78,811,245 | 82,502,244 |
| 5 | 82,629,245 | 83,208,244 |
| 5 | 83,832,245 | 88,990,244 |
| 5 | 90,024,245 | 94,814,244 |
| 5 | 108,170,081 | 108,692,472 |
| 5 | 161,943,351 | 162,023,163 |
| 5 | 162,944,423 | 166,574,681 |
| 5 | 167,884,794 | 167,911,586 |
| 5 | 177,558,395 | 177,757,394 |
| 5 | 178,897,395 | 179,047,394 |
| 6 | 119,528,302 | 120,008,301 |
| 6 | 148,638,806 | 148,677,715 |
| 6 | 150,968,308 | 151,374,307 |
| 6 | 152,826,425 | 152,958,581 |
| 6 | 154,920,290 | 154,937,005 |
| 6 | 162,700,011 | 162,990,010 |
| 6 | 165,845,616 | 166,630,010 |
| 6 | 168,287,152 | 168,387,151 |
| 6 | 169,035,141 | 171,055,067 |
| 8 | 10,002 | 2,257,558 |
| 8 | 3,770,593 | 4,031,592 |
| 8 | 6,283,593 | 29,153,081 |
| 8 | 30,504,459 | 30,690,458 |
| 9 | 21,857,763 | 21,995,242 |
| 9 | 133,617,180 | 134,057,179 |
| 9 | 139,280,180 | 141,153,179 |
| 10 | 89,717,633 | 89,720,125 |
| 13 | 36,052,001 | 36,182,000 |
| 13 | 36,226,467 | 36,267,223 |
| 13 | 36,325,705 | 36,351,723 |
| 13 | 37,491,639 | 37,586,331 |
| 13 | 39,913,628 | 39,937,395 |
| 13 | 41,182,001 | 41,502,000 |
| 13 | 47,606,656 | 47,689,357 |
| 13 | 48,982,000 | 49,059,999 |
| 13 | 50,139,493 | 50,189,656 |
| 13 | 50,502,000 | 50,866,999 |
| 13 | 79,380,817 | 82,309,215 |
| 13 | 98,922,677 | 98,930,606 |
| 13 | 104,411,846 | 104,423,574 |
| 13 | 106,154,000 | 106,193,999 |
| 13 | 110,906,662 | 110,992,890 |
| 13 | 112,172,000 | 115,101,898 |
| 15 | 33,428,709 | 33,746,708 |
| 15 | 34,127,612 | 39,112,458 |
| 15 | 42,042,039 | 42,107,044 |
| 15 | 74,763,882 | 84,405,574 |
| 15 | 88,947,581 | 89,060,042 |
| 15 | 89,717,438 | 89,849,554 |
| 15 | 90,480,658 | 90,507,150 |
| 15 | 100,888,478 | 101,045,477 |
| 16 | 2,709,458 | 2,791,999 |
| 16 | 61,902,724 | 62,079,202 |
| 16 | 80,787,059 | 80,834,676 |
| 16 | 83,064,905 | 83,109,044 |
| 16 | 86,407,653 | 86,525,478 |
| 16 | 89,151,437 | 90,027,626 |
| 17 | 2 | 153,034 |
| 17 | 11,004,276 | 13,029,876 |
| 17 | 15,059,499 | 15,061,349 |
| 17 | 16,095,276 | 17,793,275 |
| 17 | 19,348,127 | 21,049,408 |
| 17 | 19,987,323 | 19,998,261 |
| 17 | 22,167,699 | 22,198,806 |
| 17 | 29,402,462 | 29,455,342 |
| 17 | 29,505,828 | 29,845,719 |
| 17 | 37,855,171 | 37,882,923 |
| 17 | 38,216,744 | 38,245,943 |
| 17 | 49,925,002 | 49,957,001 |
| 17 | 50,439,790 | 51,853,144 |
| 17 | 69,983,097 | 70,029,709 |
| 17 | 72,724,406 | 72,835,405 |
| 18 | 67,515,021 | 68,046,048 |
| 18 | 68,217,021 | 68,651,020 |
| 19 | 60,001 | 482,000 |
| 19 | 2,339,001 | 6,800,000 |
| 19 | 57,571,189 | 57,618,188 |
| 22 | 29,314,001 | 29,436,000 |
| 22 | 38,065,429 | 38,232,054 |
| 22 | 40,832,256 | 41,043,415 |
| 22 | 45,123,794 | 45,136,770 |
| 22 | 46,901,337 | 47,151,336 |
| 22 | 48,526,246 | 48,527,972 |
| 22 | 49,059,565 | 51,203,134 |
| X | 342,776 | 2,057,013 |
| X | 3,120,001 | 3,820,000 |
| X | 4,180,001 | 6,035,000 |
| X | 23,654,080 | 23,980,079 |
| X | 28,146,879 | 28,190,728 |
| X | 32,126,986 | 32,193,324 |
| X | 32,455,483 | 32,578,048 |
| X | 32,670,180 | 32,960,708 |
| X | 32,973,080 | 33,110,079 |
| X | 36,472,700 | 36,507,763 |
| X | 40,964,807 | 44,141,105 |
| X | 44,637,009 | 44,652,047 |
| X | 44,836,029 | 44,910,420 |
| X | 65,040,476 | 67,731,292 |
| X | 80,230,743 | 80,695,975 |
| X | 82,939,429 | 85,730,297 |
| X | 86,404,345 | 86,474,844 |
| X | 88,684,216 | 88,707,930 |
| X | 107,211,858 | 107,231,075 |
| X | 113,172,996 | 115,353,791 |
| X | 123,119,812 | 123,146,892 |

**Table S3. Genes in sequencing capture**

| **Classification** | **Gene** | **Gene ID** | | | **Location** |
| --- | --- | --- | --- | --- | --- |
|  |  | **HGNC** | **Entrez** | **Ensembl** |  |
| Cancer Gene | *BRCA1* | 1100 | 672 | ENSG00000012048 | 17q21.31 |
| Cancer Gene | *BRCA2* | 1101 | 675 | ENSG00000139618 | 13q13.1 |
| Cancer Gene | *TP53* | 11998 | 7157 | ENSG00000141510 | 17p13.1 |
| Candidate TSG | *ABCA2* | 32 | 20 | ENSG00000107331 | 9q34.3 |
| Candidate TSG | *ABHD2* | 18717 | 11057 | ENSG00000140526 | 15q26.1 |
| Candidate TSG | *ABL1* | 76 | 25 | ENSG00000097007 | 9q34.12 |
| Candidate TSG | *ACER1* | 18356 | 125981 | ENSG00000167769 | 19p13.3 |
| Candidate TSG | *ACOT12* | 24436 | 134526 | ENSG00000172497 | 5q14.1 |
| Candidate TSG | *ACOT7* | 24157 | 11332 | ENSG00000097021 | 1p36.31 |
| Candidate TSG | *ACOT9* | 17152 | 23597 | ENSG00000123130 | Xp22.11 |
| Candidate TSG | *ACR* | 126 | 49 | ENSG00000100312 | 22q13.33 |
| Candidate TSG | *ACSBG1* | 29567 | 23205 | ENSG00000103740 | 15q25.1 |
| Candidate TSG | *ACSBG2* | 24174 | 81616 | ENSG00000130377 | 19p13.3 |
| Candidate TSG | *ACSF3* | 27288 | 197322 | ENSG00000176715 | 16q24.3 |
| Candidate TSG | *ACTC1* | 143 | 70 | ENSG00000159251 | 15q14 |
| Candidate TSG | *ACTRT2* | 24026 | 140625 | ENSG00000169717 | 1p36.32 |
| Candidate TSG | *ADAM28* | 206 | 10863 | ENSG00000042980 | 8p21.2 |
| Candidate TSG | *ADAM7* | 214 | 8756 | ENSG00000069206 | 8p21.2 |
| Candidate TSG | *ADAMDEC1* | 16299 | 27299 | ENSG00000134028 | 8p21.2 |
| Candidate TSG | *ADAMTS7* | 223 | 11173 | ENSG00000136378 | 15q25.1 |
| Candidate TSG | *ADAMTSL3* | 14633 | 57188 | ENSG00000156218 | 15q25.2 |
| Candidate TSG | *ADM2* | 28898 | 79924 | ENSG00000128165 | 22q13.33 |
| Candidate TSG | *ADPRHL1* | 21303 | 113622 | ENSG00000153531 | 13q34 |
| Candidate TSG | *ADRA1A* | 277 | 148 | ENSG00000120907 | 8p21.2 |
| Candidate TSG | *AES* | 307 | 166 | ENSG00000104964 | 19p13.3 |
| Candidate TSG | *AGGF1* | 24684 | 55109 | ENSG00000164252 | 5q13.3 |
| Candidate TSG | *AGPAT2* | 325 | 10555 | ENSG00000169692 | 9q34.3 |
| Candidate TSG | *AGPAT5* | 20886 | 55326 | ENSG00000155189 | 8p23.1 |
| Candidate TSG | *AGPHD1* | 34403 | 123688 | ENSG00000188266 | 15q25.1 |
| Candidate TSG | *AGRN* | 329 | 375790 | ENSG00000188157 | 1p36.33 |
| Candidate TSG | *AGTR2* | 338 | 186 | ENSG00000180772 | Xq23 |
| Candidate TSG | *AGXT2L2* | 28249 | 85007 | ENSG00000175309 | 5q35.3 |
| Candidate TSG | *AIF1L* | 28904 | 83543 | ENSG00000126878 | 9q34.12 |
| Candidate TSG | *AIM1* | 356 | 202 | ENSG00000112297 | 6q21 |
| Candidate TSG | *AKAP10* | 368 | 11216 | ENSG00000108599 | 17p11.2 |
| Candidate TSG | *AKAP17A* | 18783 | 8227 | ENSG00000197976 | Xp22.33 |
| Candidate TSG | *AKD1* | 33814 | 221264 | ENSG00000155085 | 6q21 |
| Candidate TSG | *ALDH3A1* | 405 | 218 | ENSG00000108602 | 17p11.2 |
| Candidate TSG | *ALDH3A2* | 403 | 224 | ENSG00000072210 | 17p11.2 |
| Candidate TSG | *ALG12* | 19358 | 79087 | ENSG00000182858 | 22q13.33 |
| Candidate TSG | *ALG5* | 20266 | 29880 | ENSG00000120697 | 13q13.3 |
| Candidate TSG | *ALKBH7* | 21306 | 84266 | ENSG00000125652 | 19p13.3 |
| Candidate TSG | *AMD1* | 457 | 262 | ENSG00000123505 | 6q21 |
| Candidate TSG | *ANAPC2* | 19989 | 29882 | ENSG00000176248 | 9q34.3 |
| Candidate TSG | *ANGPT2* | 485 | 285 | ENSG00000091879 | 8p23.1 |
| Candidate TSG | *ANKRD11* | 21316 | 29123 | ENSG00000167522 | 16q24.3 |
| Candidate TSG | *ANKRD24* | 29424 | 170961 | ENSG00000089847 | 19p13.3 |
| Candidate TSG | *ANKRD32* | 25408 | 84250 | ENSG00000133302 | 5q15 |
| Candidate TSG | *ANKRD34B* | 33736 | 340120 | ENSG00000189127 | 5q14.1 |
| Candidate TSG | *ANKRD34C* | 33888 | 390616 | ENSG00000235711 | 15q25.1 |
| Candidate TSG | *ANKRD54* | 25185 | 129138 | ENSG00000100124 | 22q13.1 |
| Candidate TSG | *AP3B2* | 567 | 8120 | ENSG00000103723 | 15q25.2 |
| Candidate TSG | *APBA3* | 580 | 9546 | ENSG00000011132 | 19p13.3 |
| Candidate TSG | *APOO* | 28727 | 79135 | ENSG00000184831 | Xp22.11 |
| Candidate TSG | *APOOL* | 24009 | 139322 | ENSG00000155008 | Xq21.1 |
| Candidate TSG | *AQR* | 29513 | 9716 | ENSG00000021776 | 15q14 |
| Candidate TSG | *AR* | 644 | 367 | ENSG00000169083 | Xq12 |
| Candidate TSG | *ARHGAP44* | 29096 | 9912 | ENSG00000006740 | 17p12 |
| Candidate TSG | *ARHGEF10* | 14103 | 9639 | ENSG00000104728 | 8p23.3 |
| Candidate TSG | *ARHGEF16* | 15515 | 27237 | ENSG00000130762 | 1p36.32 |
| Candidate TSG | *ARID3B* | 14350 | 10620 | ENSG00000179361 | 15q24.1 |
| Candidate TSG | *ARMC2* | 23045 | 84071 | ENSG00000118690 | 6q21 |
| Candidate TSG | *ARNT2* | 16876 | 9915 | ENSG00000172379 | 15q25.1 |
| Candidate TSG | *ARRDC1* | 28633 | 92714 | ENSG00000197070 | 9q34.3 |
| Candidate TSG | *ARRDC3* | 29263 | 57561 | ENSG00000113369 | 5q14.3 |
| Candidate TSG | *ARRDC5* | 31407 | 645432 | ENSG00000205784 | 19p13.3 |
| Candidate TSG | *ARSA* | 713 | 410 | ENSG00000100299 | 22q13.33 |
| Candidate TSG | *ASAH1* | 735 | 427 | ENSG00000104763 | 8p22 |
| Candidate TSG | *ASCC3* | 18697 | 10973 | ENSG00000112249 | 6q16.3 |
| Candidate TSG | *ASF1A* | 20995 | 25842 | ENSG00000111875 | 6q22.31 |
| Candidate TSG | *ASMT* | 750 | 438 | ENSG00000196433 | Xp22.33 |
| Candidate TSG | *ASMTL* | 751 | 8623 | ENSG00000169093 | Xp22.33 |
| Candidate TSG | *ATCAY* | 779 | 85300 | ENSG00000167654 | 19p13.3 |
| Candidate TSG | *ATG10* | 20315 | 83734 | ENSG00000152348 | 5q14.1 |
| Candidate TSG | *ATG5* | 589 | 9474 | ENSG00000057663 | 6q21 |
| Candidate TSG | *ATP11A* | 13552 | 23250 | ENSG00000068650 | 13q34 |
| Candidate TSG | *ATP4B* | 820 | 496 | ENSG00000186009 | 13q34 |
| Candidate TSG | *ATP6AP1L* | 28091 | 92270 | ENSG00000205464 | 5q14.2 |
| Candidate TSG | *ATP6V1B2* | 854 | 526 | ENSG00000147416 | 8p21.3 |
| Candidate TSG | *ATPBD4* | 30543 | 89978 | ENSG00000134146 | 15q14 |
| Candidate TSG | *AVEN* | 13509 | 57099 | ENSG00000169857 | 15q14 |
| Candidate TSG | *BCL2A1* | 991 | 597 | ENSG00000140379 | 15q25.1 |
| Candidate TSG | *BEND3* | 23040 | 57673 | ENSG00000178409 | 6q21 |
| Candidate TSG | *BET3L* | 21090 | 100128327 | ENSG00000173626 | 6q22.1 |
| Candidate TSG | *BIN3* | 1054 | 55909 | ENSG00000147439 | 8p21.3 |
| Candidate TSG | *BLK* | 1057 | 640 | ENSG00000136573 | 8p23.1 |
| Candidate TSG | *BMP1* | 1067 | 649 | ENSG00000168487 | 8p21.3 |
| Candidate TSG | *BNC1* | 1081 | 646 | ENSG00000169594 | 15q25.2 |
| Candidate TSG | *BNIP3L* | 1085 | 665 | ENSG00000104765 | 8p21.2 |
| Candidate TSG | *BRD1* | 1102 | 23774 | ENSG00000100425 | 22q13.33 |
| Candidate TSG | *BRD7P3* | 24171 | 23629 | ENSG00000169075 | 6q22.31 |
| Candidate TSG | *BTBD1* | 1120 | 53339 | ENSG00000064726 | 15q25.2 |
| Candidate TSG | *BVES* | 1152 | 11149 | ENSG00000112276 | 6q21 |
| Candidate TSG | *C13orf35* | 33793 | 400165 | ENSG00000197595 | 13q34 |
| Candidate TSG | *C15orf17* | 24695 | 57184 | ENSG00000178761 | 15q24.1 |
| Candidate TSG | *C15orf24* | 24301 | 56851 | ENSG00000134153 | 15q14 |
| Candidate TSG | *C15orf26* | 26782 | 161502 | ENSG00000156206 | 15q25.1 |
| Candidate TSG | *C15orf27* | 26763 | 123591 | ENSG00000169758 | 15q24.2 |
| Candidate TSG | *C15orf29* | 26199 | 79768 | ENSG00000134152 | 15q14 |
| Candidate TSG | *C15orf37* | 27521 | 283687 | ENSG00000223712 | 15q25.1 |
| Candidate TSG | *C15orf39* | 24497 | 56905 | ENSG00000167173 | 15q24.2 |
| Candidate TSG | *C15orf40* | 28443 | 123207 | ENSG00000169609 | 15q25.2 |
| Candidate TSG | *C15orf41* | 26929 | 84529 | ENSG00000186073 | 15q14 |
| Candidate TSG | *C15orf53* | 33796 | 400359 | ENSG00000175779 | 15q14 |
| Candidate TSG | *C15orf55* | 29919 | 256646 | ENSG00000184507 | 15q14 |
| Candidate TSG | *C16orf55* | 26463 | 124045 | ENSG00000167523 | 16q24.3 |
| Candidate TSG | *C16orf7* | 13526 | 9605 | ENSG00000075399 | 16q24.3 |
| Candidate TSG | *C17orf76* | 32403 | 388341 | ENSG00000181350 | 17p11.2 |
| Candidate TSG | *C17orf76-AS1* | 28619 | 125144 | ENSG00000175061 | 17p11.2 |
| Candidate TSG | *C19orf10* | 16948 | 56005 | ENSG00000074842 | 19p13.3 |
| Candidate TSG | *C19orf28* | 28299 | 126321 | ENSG00000161091 | 19p13.3 |
| Candidate TSG | *C19orf29* | 29938 | 58509 | ENSG00000105298 | 19p13.3 |
| Candidate TSG | *C19orf70* | 33702 | 125988 | ENSG00000174917 | 19p13.3 |
| Candidate TSG | *C19orf71* | 34496 | 100128569 | ENSG00000183397 | 19p13.3 |
| Candidate TSG | *C19orf77* | 37244 | 284422 | ENSG00000095932 | 19p13.3 |
| Candidate TSG | *C1orf159* | 26062 | 54991 | ENSG00000131591 | 1p36.33 |
| Candidate TSG | *C1orf222* | 27917 | 85452 | ENSG00000142609 | 1p36.33 |
| Candidate TSG | *C1orf86* | 26428 | 199990 | ENSG00000162585 | 1p36.33 |
| Candidate TSG | *C1orf93* | 28390 | 127281 | ENSG00000157870 | 1p36.32 |
| Candidate TSG | *C22orf41* | 35245 | 644186 | ENSG00000217442 | 22q13.33 |
| Candidate TSG | *C22orf42* | 27160 | 150297 | ENSG00000205856 | 22q12.3 |
| Candidate TSG | *C2CD4C* | 29417 | 126567 | ENSG00000183186 | 19p13.3 |
| Candidate TSG | *C3* | 1318 | 718 | ENSG00000125730 | 19p13.3 |
| Candidate TSG | *C6orf120* | 21247 | 387263 | ENSG00000185127 | 6q27 |
| Candidate TSG | *C6orf168* | 20742 | 84553 | ENSG00000146267 | 6q16.2 |
| Candidate TSG | *C6orf186* | 21566 | 728464 | ENSG00000053328 | 6q21 |
| Candidate TSG | *C6orf203* | 17971 | 51250 | ENSG00000130349 | 6q21 |
| Candidate TSG | *C6orf204* | 21638 | 387119 | ENSG00000111860 | 6q22.31 |
| Candidate TSG | *C6orf225* | 33858 | 619208 | ENSG00000203778 | 6q21 |
| Candidate TSG | *C6orf70* | 21056 | 55780 | ENSG00000130023 | 6q27 |
| Candidate TSG | *C8G* | 1354 | 733 | ENSG00000176919 | 9q34.3 |
| Candidate TSG | *C8orf42* | 26951 | 157695 | ENSG00000180190 | 8p23.3 |
| Candidate TSG | *C8orf48* | 26345 | 157773 | ENSG00000164743 | 8p22 |
| Candidate TSG | *C8orf58* | 32233 | 541565 | ENSG00000241852 | 8p21.3 |
| Candidate TSG | *C8orf74* | 32296 | 203076 | ENSG00000171060 | 8p23.1 |
| Candidate TSG | *C8orf80* | 33550 | 389643 | ENSG00000189233 | 8p21.1 |
| Candidate TSG | *C9orf139* | 31426 | 401563 | ENSG00000180539 | 9q34.3 |
| Candidate TSG | *C9orf140* | 28055 | 89958 | ENSG00000186193 | 9q34.3 |
| Candidate TSG | *C9orf142* | 27849 | 286257 | ENSG00000148362 | 9q34.3 |
| Candidate TSG | *C9orf163* | 26718 | 158055 | ENSG00000196366 | 9q34.3 |
| Candidate TSG | *C9orf167* | 25981 | 54863 | ENSG00000198113 | 9q34.3 |
| Candidate TSG | *C9orf169* | 30529 | 375791 | ENSG00000197191 | 9q34.3 |
| Candidate TSG | *C9orf172* | 37284 | 389813 | ENSG00000232434 | 9q34.3 |
| Candidate TSG | *C9orf173* | 37285 | 441476 | ENSG00000197768 | 9q34.3 |
| Candidate TSG | *C9orf37* | 23395 | 85026 | ENSG00000203993 | 9q34.3 |
| Candidate TSG | *C9orf86* | 24703 | 55684 | ENSG00000196642 | 9q34.3 |
| Candidate TSG | *CA10* | 1369 | 56934 | ENSG00000154975 | 17q22 |
| Candidate TSG | *CACNA1B* | 1389 | 774 | ENSG00000148408 | 9q34.3 |
| Candidate TSG | *CALML6* | 24193 | 163688 | ENSG00000169885 | 1p36.33 |
| Candidate TSG | *CAMK2N1* | 24190 | 55450 | ENSG00000162545 | 1p36.12 |
| Candidate TSG | *CAMTA1* | 18806 | 23261 | ENSG00000171735 | 1p36.31 |
| Candidate TSG | *CAPS* | 1487 | 828 | ENSG00000105519 | 19p13.3 |
| Candidate TSG | *CASK* | 1497 | 8573 | ENSG00000147044 | Xp11.4 |
| Candidate TSG | *CCDC144A* | 29072 | 9720 | ENSG00000170160 | 17p11.2 |
| Candidate TSG | *CCDC144NL* | 33735 | 339184 | ENSG00000205212 | 17p11.2 |
| Candidate TSG | *CCDC25* | 25591 | 55246 | ENSG00000147419 | 8p21.1 |
| Candidate TSG | *CCDC94* | 25518 | 55702 | ENSG00000105248 | 19p13.3 |
| Candidate TSG | *CCNC* | 1581 | 892 | ENSG00000112237 | 6q16.2 |
| Candidate TSG | *CCNH* | 1594 | 902 | ENSG00000134480 | 5q14.3 |
| Candidate TSG | *CD164* | 1632 | 8763 | ENSG00000135535 | 6q21 |
| Candidate TSG | *CD226* | 16961 | 10666 | ENSG00000150637 | 18q22.2 |
| Candidate TSG | *CD70* | 11937 | 970 | ENSG00000125726 | 19p13.3 |
| Candidate TSG | *CDC16* | 1720 | 8881 | ENSG00000130177 | 13q34 |
| Candidate TSG | *CDC40* | 17350 | 51362 | ENSG00000168438 | 6q21 |
| Candidate TSG | *CDCA2* | 14623 | 157313 | ENSG00000184661 | 8p21.2 |
| Candidate TSG | *CDH13* | 1753 | 1012 | ENSG00000140945 | 16q23.3 |
| Candidate TSG | *CDH15* | 1754 | 1013 | ENSG00000129910 | 16q24.3 |
| Candidate TSG | *CDH8* | 1767 | 1006 | ENSG00000150394 | 16q21 |
| Candidate TSG | *CDK10* | 1770 | 8558 | ENSG00000185324 | 16q24.3 |
| Candidate TSG | *CDK19* | 19338 | 23097 | ENSG00000155111 | 6q21 |
| Candidate TSG | *CDKN2A* | 1787 | 1029 | ENSG00000147889 | 9p21.3 |
| Candidate TSG | *CDYL2* | 23030 | 124359 | ENSG00000166446 | 16q23.2 |
| Candidate TSG | *CELF5* | 14058 | 60680 | ENSG00000161082 | 19p13.3 |
| Candidate TSG | *CELSR1* | 1850 | 9620 | ENSG00000075275 | 22q13.31 |
| Candidate TSG | *CENPV* | 29920 | 201161 | ENSG00000166582 | 17p11.2 |
| Candidate TSG | *CEP57L1* | 21561 | 285753 | ENSG00000183137 | 6q21 |
| Candidate TSG | *CERK* | 19256 | 64781 | ENSG00000100422 | 22q13.31 |
| Candidate TSG | *CERS3* | 23752 | 204219 | ENSG00000154227 | 15q26.3 |
| Candidate TSG | *CHAF1A* | 1910 | 10036 | ENSG00000167670 | 19p13.3 |
| Candidate TSG | *CHD5* | 16816 | 26038 | ENSG00000116254 | 1p36.31 |
| Candidate TSG | *CHKB* | 1938 | 1120 | ENSG00000100288 | 22q13.33 |
| Candidate TSG | *CHM* | 1940 | 1121 | ENSG00000188419 | Xq21.2 |
| Candidate TSG | *CHMP1A* | 8740 | 5119 | ENSG00000131165 | 16q24.3 |
| Candidate TSG | *CHMP7* | 28439 | 91782 | ENSG00000147457 | 8p21.3 |
| Candidate TSG | *CHRM5* | 1954 | 1133 | ENSG00000184984 | 15q14 |
| Candidate TSG | *CHRNA2* | 1956 | 1135 | ENSG00000120903 | 8p21.2 |
| Candidate TSG | *CHRNA3* | 1957 | 1136 | ENSG00000080644 | 15q25.1 |
| Candidate TSG | *CHRNA5* | 1959 | 1138 | ENSG00000169684 | 15q25.1 |
| Candidate TSG | *CHRNB4* | 1964 | 1143 | ENSG00000117971 | 15q25.1 |
| Candidate TSG | *CIB2* | 24579 | 10518 | ENSG00000136425 | 15q25.1 |
| Candidate TSG | *CKMT2* | 1996 | 1160 | ENSG00000131730 | 5q14.1 |
| Candidate TSG | *CLDN23* | 17591 | 137075 | ENSG00000253958 | 8p23.1 |
| Candidate TSG | *CLIC3* | 2064 | 9022 | ENSG00000169583 | 9q34.3 |
| Candidate TSG | *CLK3* | 2071 | 1198 | ENSG00000179335 | 15q24.1 |
| Candidate TSG | *CLN8* | 2079 | 2055 | ENSG00000182372 | 8p23.3 |
| Candidate TSG | *CLPP* | 2084 | 8192 | ENSG00000125656 | 19p13.3 |
| Candidate TSG | *CLU* | 2095 | 1191 | ENSG00000120885 | 8p21.1 |
| Candidate TSG | *CMYA5* | 14305 | 202333 | ENSG00000164309 | 5q14.1 |
| Candidate TSG | *CNOT7* | 14101 | 29883 | ENSG00000198791 | 8p22 |
| Candidate TSG | *COBRA1* | 24324 | 25920 | ENSG00000188986 | 9q34.3 |
| Candidate TSG | *COL10A1* | 2185 | 1300 | ENSG00000123500 | 6q22.1 |
| Candidate TSG | *COL23A1* | 22990 | 91522 | ENSG00000050767 | 5q35.3 |
| Candidate TSG | *COL4A1* | 2202 | 1282 | ENSG00000187498 | 13q34 |
| Candidate TSG | *COL4A2* | 2203 | 1284 | ENSG00000134871 | 13q34 |
| Candidate TSG | *COMMD4* | 26027 | 54939 | ENSG00000140365 | 15q24.2 |
| Candidate TSG | *COPS3* | 2239 | 8533 | ENSG00000141030 | 17p11.2 |
| Candidate TSG | *COQ3* | 18175 | 51805 | ENSG00000132423 | 6q16.2 |
| Candidate TSG | *COX5A* | 2267 | 9377 | ENSG00000178741 | 15q24.2 |
| Candidate TSG | *COX7C* | 2292 | 1350 | ENSG00000127184 | 5q14.3 |
| Candidate TSG | *CPEB1* | 21744 | 64506 | ENSG00000214575 | 15q25.2 |
| Candidate TSG | *CPLX3* | 27652 | 594855 | ENSG00000213578 | 15q24.1 |
| Candidate TSG | *CPNE7* | 2320 | 27132 | ENSG00000178773 | 16q24.3 |
| Candidate TSG | *CPT1B* | 2329 | 1375 | ENSG00000205560 | 22q13.33 |
| Candidate TSG | *CRABP1* | 2338 | 1381 | ENSG00000166426 | 15q25.1 |
| Candidate TSG | *CRB3* | 20237 | 92359 | ENSG00000130545 | 19p13.3 |
| Candidate TSG | *CREB3L3* | 18855 | 84699 | ENSG00000060566 | 19p13.3 |
| Candidate TSG | *CRELD2* | 28150 | 79174 | ENSG00000184164 | 22q13.33 |
| Candidate TSG | *CRHBP* | 2356 | 1393 | ENSG00000145708 | 5q13.3 |
| Candidate TSG | *CRLF2* | 14281 | 64109 | ENSG00000205755 | Xp22.33 |
| Candidate TSG | *CSF2RA* | 2435 | 1438 | ENSG00000198223 | Xp22.33 |
| Candidate TSG | *CSGALNACT1* | 24290 | 55790 | ENSG00000147408 | 8p21.3 |
| Candidate TSG | *CSK* | 2444 | 1445 | ENSG00000103653 | 15q24.1 |
| Candidate TSG | *CSPG4* | 2466 | 1464 | ENSG00000173546 | 15q24.2 |
| Candidate TSG | *CTSB* | 2527 | 1508 | ENSG00000164733 | 8p23.1 |
| Candidate TSG | *CTSH* | 2535 | 1512 | ENSG00000103811 | 15q25.1 |
| Candidate TSG | *CUL4A* | 2554 | 8451 | ENSG00000139842 | 13q34 |
| Candidate TSG | *CXorf58* | 26356 | 254158 | ENSG00000165182 | Xp22.11 |
| Candidate TSG | *CYLC1* | 2582 | 1538 | ENSG00000183035 | Xq21.1 |
| Candidate TSG | *CYP1A1* | 2595 | 1543 | ENSG00000140465 | 15q24.1 |
| Candidate TSG | *CYP1A2* | 2596 | 1544 | ENSG00000140505 | 15q24.1 |
| Candidate TSG | *DACH2* | 16814 | 117154 | ENSG00000126733 | Xq21.2 |
| Candidate TSG | *DAPK3* | 2676 | 1613 | ENSG00000167657 | 19p13.3 |
| Candidate TSG | *DCBLD1* | 21479 | 285761 | ENSG00000164465 | 6q22.1 |
| Candidate TSG | *DCLK1* | 2700 | 9201 | ENSG00000133083 | 13q13.3 |
| Candidate TSG | *DCUN1D2* | 20328 | 55208 | ENSG00000150401 | 13q34 |
| Candidate TSG | *DDO* | 2727 | 8528 | ENSG00000203797 | 6q21 |
| Candidate TSG | *DEF8* | 25969 | 54849 | ENSG00000140995 | 16q24.3 |
| Candidate TSG | *DEFA1* | 2761 | 1667 | ENSG00000206047 | 8p23.1 |
| Candidate TSG | *DEFA1B* | 33596 | 728358 | ENSG00000240247 | 8p23.1 |
| Candidate TSG | *DEFA3* | 2762 | 1668 | ENSG00000239839 | 8p23.1 |
| Candidate TSG | *DEFA4* | 2763 | 1669 | ENSG00000164821 | 8p23.1 |
| Candidate TSG | *DEFA5* | 2764 | 1670 | ENSG00000164816 | 8p23.1 |
| Candidate TSG | *DEFA6* | 2765 | 1671 | ENSG00000164822 | 8p23.1 |
| Candidate TSG | *DEFB1* | 2766 | 1672 | ENSG00000164825 | 8p23.1 |
| Candidate TSG | *DEFB103A* | 15967 | 414325 | ENSG00000176797 | 8p23.1 |
| Candidate TSG | *DEFB103B* | 31702 | 55894 | ENSG00000177243 | 8p23.1 |
| Candidate TSG | *DEFB104A* | 18115 | 140596 | ENSG00000176782 | 8p23.1 |
| Candidate TSG | *DEFB104B* | 26165 | 503618 | ENSG00000177023 | 8p23.1 |
| Candidate TSG | *DEFB105A* | 18087 | 245908 | ENSG00000186562 | 8p23.1 |
| Candidate TSG | *DEFB105B* | 29930 | 504180 | ENSG00000186599 | 8p23.1 |
| Candidate TSG | *DEFB106A* | 18088 | 245909 | ENSG00000186579 | 8p23.1 |
| Candidate TSG | *DEFB106B* | 28879 | 503841 | ENSG00000187082 | 8p23.1 |
| Candidate TSG | *DEFB107A* | 18086 | 245910 | ENSG00000186572 | 8p23.1 |
| Candidate TSG | *DEFB107B* | 31918 | 503614 | ENSG00000198129 | 8p23.1 |
| Candidate TSG | *DEFB130* | 18107 | 245940 | ENSG00000232948 | 8p23.1 |
| Candidate TSG | *DEFB134* | 32399 | 613211 | ENSG00000205882 | 8p23.1 |
| Candidate TSG | *DEFB135* | 32400 | 613209 | ENSG00000205883 | 8p23.1 |
| Candidate TSG | *DEFB136* | 34433 | 613210 | ENSG00000205884 | 8p23.1 |
| Candidate TSG | *DEFB4A* | 2767 | 1673 | ENSG00000171711 | 8p23.1 |
| Candidate TSG | *DENND1C* | 26225 | 79958 | ENSG00000205744 | 19p13.3 |
| Candidate TSG | *DET1* | 25477 | 55070 | ENSG00000140543 | 15q25.3 |
| Candidate TSG | *DHFR* | 2861 | 1719 | ENSG00000228716 | 5q14.1 |
| Candidate TSG | *DHRS7B* | 24547 | 25979 | ENSG00000109016 | 17p11.2 |
| Candidate TSG | *DIRAS1* | 19127 | 148252 | ENSG00000176490 | 19p13.3 |
| Candidate TSG | *DLC1* | 2897 | 10395 | ENSG00000164741 | 8p22 |
| Candidate TSG | *DLL1* | 2908 | 28514 | ENSG00000198719 | 6q27 |
| Candidate TSG | *DMD* | 2928 | 1756 | ENSG00000198947 | Xp21.1 |
| Candidate TSG | *DNAH9* | 2953 | 1770 | ENSG00000007174 | 17p12 |
| Candidate TSG | *DNAJA4* | 14885 | 55466 | ENSG00000140403 | 15q25.1 |
| Candidate TSG | *DNAJC11* | 25570 | 55735 | ENSG00000007923 | 1p36.31 |
| Candidate TSG | *DOCK5* | 23476 | 80005 | ENSG00000147459 | 8p21.2 |
| Candidate TSG | *DOHH* | 28662 | 83475 | ENSG00000129932 | 19p13.3 |
| Candidate TSG | *DOK2* | 2991 | 9046 | ENSG00000147443 | 8p21.3 |
| Candidate TSG | *DOK6* | 28301 | 220164 | ENSG00000206052 | 18q22.2 |
| Candidate TSG | *DPEP1* | 3002 | 1800 | ENSG00000015413 | 16q24.3 |
| Candidate TSG | *DPP7* | 14892 | 29952 | ENSG00000176978 | 9q34.3 |
| Candidate TSG | *DPP9* | 18648 | 91039 | ENSG00000142002 | 19p13.3 |
| Candidate TSG | *DPYSL2* | 3014 | 1808 | ENSG00000092964 | 8p21.2 |
| Candidate TSG | *DSE* | 21144 | 29940 | ENSG00000111817 | 6q22.1 |
| Candidate TSG | *DUS3L* | 26920 | 56931 | ENSG00000141994 | 19p13.3 |
| Candidate TSG | *DUX4* | 3082 | 22947 | ENSG00000258389 | 4q35.2 |
| Candidate TSG | *DUX4L2* | 37267 | 728410 | ENSG00000259128 | 4q35.2 |
| Candidate TSG | *DUX4L5* | 38689 | 653545 | ENSG00000227476 | 4q35.2 |
| Candidate TSG | *DUX4L6* | 37265 | 653544 | ENSG00000228188 | 4q35.2 |
| Candidate TSG | *DUX4L7* | 37266 | 653543 | ENSG00000237516 | 4q35.2 |
| Candidate TSG | *EBF2* | 19090 | 64641 | ENSG00000221818 | 8p21.2 |
| Candidate TSG | *EBI3* | 3129 | 10148 | ENSG00000105246 | 19p13.3 |
| Candidate TSG | *EDA2R* | 17756 | 60401 | ENSG00000131080 | Xq12 |
| Candidate TSG | *EDC3* | 26114 | 80153 | ENSG00000179151 | 15q24.1 |
| Candidate TSG | *EDF1* | 3164 | 8721 | ENSG00000107223 | 9q34.3 |
| Candidate TSG | *EEF2* | 3214 | 1938 | ENSG00000167658 | 19p13.3 |
| Candidate TSG | *EFHA2* | 27820 | 286097 | ENSG00000155970 | 8p22 |
| Candidate TSG | *EFHC2* | 26233 | 80258 | ENSG00000183690 | Xp11.3 |
| Candidate TSG | *EFTUD1* | 25789 | 79631 | ENSG00000140598 | 15q25.2 |
| Candidate TSG | *EGFL7* | 20594 | 51162 | ENSG00000172889 | 9q34.3 |
| Candidate TSG | *EGR3* | 3240 | 1960 | ENSG00000179388 | 8p21.3 |
| Candidate TSG | *EHMT1* | 24650 | 79813 | ENSG00000181090 | 9q34.3 |
| Candidate TSG | *ELAC2* | 14198 | 60528 | ENSG00000006744 | 17p12 |
| Candidate TSG | *ELP3* | 20696 | 55140 | ENSG00000134014 | 8p21.1 |
| Candidate TSG | *ENTPD2* | 3364 | 954 | ENSG00000054179 | 9q34.3 |
| Candidate TSG | *ENTPD4* | 14573 | 9583 | ENSG00000197217 | 8p21.2 |
| Candidate TSG | *ENTPD8* | 24860 | 377841 | ENSG00000188833 | 9q34.3 |
| Candidate TSG | *EPB49* | 3382 | 2039 | ENSG00000158856 | 8p21.3 |
| Candidate TSG | *EPHX2* | 3402 | 2053 | ENSG00000120915 | 8p21.2 |
| Candidate TSG | *ERBB2* | 3430 | 2064 | ENSG00000141736 | 17q12 |
| Candidate TSG | *ERI1* | 23994 | 90459 | ENSG00000104626 | 8p23.1 |
| Candidate TSG | *ERICH1* | 27234 | 157697 | ENSG00000104714 | 8p23.3 |
| Candidate TSG | *ESCO2* | 27230 | 157570 | ENSG00000171320 | 8p21.1 |
| Candidate TSG | *ESPN* | 13281 | 83715 | ENSG00000187017 | 1p36.31 |
| Candidate TSG | *ETFA* | 3481 | 2108 | ENSG00000140374 | 15q24.3 |
| Candidate TSG | *EVI2A* | 3499 | 2123 | ENSG00000126860 | 17q11.2 |
| Candidate TSG | *EVI2B* | 3500 | 2124 | ENSG00000185862 | 17q11.2 |
| Candidate TSG | *EXD3* | 26023 | 54932 | ENSG00000187609 | 9q34.3 |
| Candidate TSG | *EXOSC8* | 17035 | 11340 | ENSG00000120699 | 13q13.3 |
| Candidate TSG | *EXTL3* | 3518 | 2137 | ENSG00000012232 | 8p21.1 |
| Candidate TSG | *F10* | 3528 | 2159 | ENSG00000126218 | 13q34 |
| Candidate TSG | *F2R* | 3537 | 2149 | ENSG00000181104 | 5q13.3 |
| Candidate TSG | *F2RL1* | 3538 | 2150 | ENSG00000164251 | 5q13.3 |
| Candidate TSG | *F2RL2* | 3539 | 2151 | ENSG00000164220 | 5q13.3 |
| Candidate TSG | *F7* | 3544 | 2155 | ENSG00000057593 | 13q34 |
| Candidate TSG | *FAH* | 3579 | 2184 | ENSG00000103876 | 15q25.1 |
| Candidate TSG | *FAM103A1* | 31022 | 83640 | ENSG00000169612 | 15q25.2 |
| Candidate TSG | *FAM108C1* | 26925 | 58489 | ENSG00000136379 | 15q25.1 |
| Candidate TSG | *FAM116B* | 32690 | 414918 | ENSG00000205593 | 22q13.33 |
| Candidate TSG | *FAM120B* | 21109 | 84498 | ENSG00000112584 | 6q27 |
| Candidate TSG | *FAM151B* | 33716 | 167555 | ENSG00000152380 | 5q14.1 |
| Candidate TSG | *FAM154B* | 33727 | 283726 | ENSG00000188659 | 15q25.2 |
| Candidate TSG | *FAM157A* | 34079 | 100132403 | ENSG00000233013 | 9q34.3 |
| Candidate TSG | *FAM160B2* | 16492 | 64760 | ENSG00000158863 | 8p21.3 |
| Candidate TSG | *FAM162B* | 21549 | 221303 | ENSG00000183807 | 6q22.1 |
| Candidate TSG | *FAM166A* | 33818 | 401565 | ENSG00000188163 | 9q34.3 |
| Candidate TSG | *FAM167A* | 15549 | 83648 | ENSG00000154319 | 8p23.1 |
| Candidate TSG | *FAM172A* | 25365 | 83989 | ENSG00000113391 | 5q15 |
| Candidate TSG | *FAM184A* | 20991 | 79632 | ENSG00000111879 | 6q22.31 |
| Candidate TSG | *FAM26D* | 21094 | 221301 | ENSG00000164451 | 6q22.1 |
| Candidate TSG | *FAM26E* | 21568 | 254228 | ENSG00000178033 | 6q22.1 |
| Candidate TSG | *FAM26F* | 33391 | 441168 | ENSG00000188820 | 6q22.1 |
| Candidate TSG | *FAM43B* | 31791 | 163933 | ENSG00000183114 | 1p36.12 |
| Candidate TSG | *FAM48A* | 20596 | 55578 | ENSG00000102710 | 13q13.3 |
| Candidate TSG | *FAM69B* | 28290 | 138311 | ENSG00000165716 | 9q34.3 |
| Candidate TSG | *FAM70B* | 28297 | 348013 | ENSG00000184497 | 13q34 |
| Candidate TSG | *FAM81B* | 26335 | 153643 | ENSG00000153347 | 5q15 |
| Candidate TSG | *FAM86B1* | 28268 | 85002 | ENSG00000186523 | 8p23.1 |
| Candidate TSG | *FAM86B2* | 32222 | 653333 | ENSG00000145002 | 8p23.1 |
| Candidate TSG | *FAM90A7* | - | 441317 | ENSG00000236660 | 8p23.1 |
| Candidate TSG | *FAM98B* | 26773 | 283742 | ENSG00000171262 | 15q14 |
| Candidate TSG | *FANCA* | 3582 | 2175 | ENSG00000187741 | 16q24.3 |
| Candidate TSG | *FANCI* | 25568 | 55215 | ENSG00000140525 | 15q26.1 |
| Candidate TSG | *FARP1* | 3591 | 10160 | ENSG00000152767 | 13q32.2 |
| Candidate TSG | *FBXO16* | 13618 | 157574 | ENSG00000214050 | 8p21.1 |
| Candidate TSG | *FBXO22* | 13593 | 26263 | ENSG00000167196 | 15q24.2 |
| Candidate TSG | *FBXO25* | 13596 | 26260 | ENSG00000147364 | 8p23.3 |
| Candidate TSG | *FBXW5* | 13613 | 54461 | ENSG00000159069 | 9q34.3 |
| Candidate TSG | *FDFT1* | 3629 | 2222 | ENSG00000079459 | 8p23.1 |
| Candidate TSG | *FEM1A* | 16934 | 55527 | ENSG00000141965 | 19p13.3 |
| Candidate TSG | *FER* | 3655 | 2241 | ENSG00000151422 | 5q21.3 |
| Candidate TSG | *FGF17* | 3673 | 8822 | ENSG00000158815 | 8p21.3 |
| Candidate TSG | *FGF20* | 3677 | 26281 | ENSG00000078579 | 8p22 |
| Candidate TSG | *FGL1* | 3695 | 2267 | ENSG00000104760 | 8p22 |
| Candidate TSG | *FIBCD1* | 25922 | 84929 | ENSG00000130720 | 9q34.12 |
| Candidate TSG | *FIG4* | 16873 | 9896 | ENSG00000112367 | 6q21 |
| Candidate TSG | *FLCN* | 27310 | 201163 | ENSG00000154803 | 17p11.2 |
| Candidate TSG | *FOXO1* | 3819 | 2308 | ENSG00000150907 | 13q14.11 |
| Candidate TSG | *FOXO3* | 3821 | 2309 | ENSG00000118689 | 6q21 |
| Candidate TSG | *FRG1* | 3954 | 2483 | ENSG00000109536 | 4q35.2 |
| Candidate TSG | *FRG2* | 19136 | 448831 | ENSG00000205097 | 4q35.2 |
| Candidate TSG | *FRK* | 3955 | 2444 | ENSG00000111816 | 6q22.1 |
| Candidate TSG | *FSD1* | 13745 | 79187 | ENSG00000105255 | 19p13.3 |
| Candidate TSG | *FSD2* | 18024 | 123722 | ENSG00000186628 | 15q25.2 |
| Candidate TSG | *FUT3* | 4014 | 2525 | ENSG00000171124 | 19p13.3 |
| Candidate TSG | *FUT5* | 4016 | 2527 | ENSG00000130383 | 19p13.3 |
| Candidate TSG | *FUT6* | 4017 | 2528 | ENSG00000156413 | 19p13.3 |
| Candidate TSG | *FUT7* | 4018 | 2529 | ENSG00000180549 | 9q34.3 |
| Candidate TSG | *FYN* | 4037 | 2534 | ENSG00000010810 | 6q21 |
| Candidate TSG | *FZD3* | 4041 | 7976 | ENSG00000104290 | 8p21.1 |
| Candidate TSG | *FZR1* | 24824 | 51343 | ENSG00000105325 | 19p13.3 |
| Candidate TSG | *GABRD* | 4084 | 2563 | ENSG00000187730 | 1p36.33 |
| Candidate TSG | *GADD45B* | 4096 | 4616 | ENSG00000099860 | 19p13.3 |
| Candidate TSG | *GALR3* | 4134 | 8484 | ENSG00000128310 | 22q13.1 |
| Candidate TSG | *GAS6* | 4168 | 2621 | ENSG00000183087 | 13q34 |
| Candidate TSG | *GATA4* | 4173 | 2626 | ENSG00000136574 | 8p23.1 |
| Candidate TSG | *GCAT* | 4188 | 23464 | ENSG00000100116 | 22q13.1 |
| Candidate TSG | *GFRA2* | 4244 | 2675 | ENSG00000168546 | 8p21.3 |
| Candidate TSG | *GIPC3* | 18183 | 126326 | ENSG00000179855 | 19p13.3 |
| Candidate TSG | *GJD2* | 19154 | 57369 | ENSG00000159248 | 15q14 |
| Candidate TSG | *GNA11* | 4379 | 2767 | ENSG00000088256 | 19p13.3 |
| Candidate TSG | *GNA15* | 4383 | 2769 | ENSG00000060558 | 19p13.3 |
| Candidate TSG | *GNB1* | 4396 | 2782 | ENSG00000078369 | 1p36.33 |
| Candidate TSG | *GNG7* | 4410 | 2788 | ENSG00000176533 | 19p13.3 |
| Candidate TSG | *GNRH1* | 4419 | 2796 | ENSG00000147437 | 8p21.2 |
| Candidate TSG | *GOLGA6D* | 32204 | 653643 | ENSG00000140478 | 15q24.2 |
| Candidate TSG | *GOLGA6L9* | 37229 | 440295 | ENSG00000197978 | 15q25.2 |
| Candidate TSG | *GOLGA8A* | 31972 | 23015 | ENSG00000175265 | 15q14 |
| Candidate TSG | *GOLGA8B* | 31973 | 440270 | ENSG00000215252 | 15q14 |
| Candidate TSG | *GOPC* | 17643 | 57120 | ENSG00000047932 | 6q22.1 |
| Candidate TSG | *GPR108* | 17829 | 56927 | ENSG00000125734 | 19p13.3 |
| Candidate TSG | *GPR153* | 23618 | 387509 | ENSG00000158292 | 1p36.31 |
| Candidate TSG | *GPR34* | 4490 | 2857 | ENSG00000171659 | Xp11.4 |
| Candidate TSG | *GPR6* | 4515 | 2830 | ENSG00000146360 | 6q21 |
| Candidate TSG | *GPR82* | 4533 | 27197 | ENSG00000171657 | Xp11.4 |
| Candidate TSG | *GPR98* | 17416 | 84059 | ENSG00000164199 | 5q14.3 |
| Candidate TSG | *GPRC6A* | 18510 | 222545 | ENSG00000173612 | 6q22.1 |
| Candidate TSG | *GRAMD4* | 29113 | 23151 | ENSG00000075240 | 22q13.31 |
| Candidate TSG | *GRID2* | 4576 | 2895 | ENSG00000152208 | 4q22.1 |
| Candidate TSG | *GRIK2* | 4580 | 2898 | ENSG00000164418 | 6q16.3 |
| Candidate TSG | *GRIN1* | 4584 | 2902 | ENSG00000176884 | 9q34.3 |
| Candidate TSG | *GRK1* | 10013 | 6011 | ENSG00000185974 | 13q34 |
| Candidate TSG | *GRTP1* | 20310 | 79774 | ENSG00000139835 | 13q34 |
| Candidate TSG | *GSR* | 4623 | 2936 | ENSG00000104687 | 8p12 |
| Candidate TSG | *GTF2E2* | 4651 | 2961 | ENSG00000197265 | 8p12 |
| Candidate TSG | *GTF2F1* | 4652 | 2962 | ENSG00000125651 | 19p13.3 |
| Candidate TSG | *GTF3C6* | 20872 | 112495 | ENSG00000155115 | 6q21 |
| Candidate TSG | *H1F0* | 4714 | 3005 | ENSG00000189060 | 22q13.1 |
| Candidate TSG | *HACE1* | 21033 | 57531 | ENSG00000085382 | 6q16.3 |
| Candidate TSG | *HAPLN1* | 2380 | 1404 | ENSG00000145681 | 5q14.3 |
| Candidate TSG | *HDAC10* | 18128 | 83933 | ENSG00000100429 | 22q13.33 |
| Candidate TSG | *HDAC2* | 4853 | 3066 | ENSG00000196591 | 6q21 |
| Candidate TSG | *HDGFRP2* | - | 84717 | ENSG00000167674 | 19p13.3 |
| Candidate TSG | *HDGFRP3* | - | 50810 | ENSG00000166503 | 15q25.2 |
| Candidate TSG | *HDX* | 26411 | 139324 | ENSG00000165259 | Xq21.1 |
| Candidate TSG | *HEPH* | 4866 | 9843 | ENSG00000089472 | Xq12 |
| Candidate TSG | *HES2* | 16005 | 54626 | ENSG00000069812 | 1p36.31 |
| Candidate TSG | *HES3* | 26226 | 390992 | ENSG00000173673 | 1p36.31 |
| Candidate TSG | *HES4* | 24149 | 57801 | ENSG00000188290 | 1p36.33 |
| Candidate TSG | *HES5* | 19764 | 388585 | ENSG00000197921 | 1p36.32 |
| Candidate TSG | *HGC6.3* | - | 100128124 | - | 6q27 |
| Candidate TSG | *HMBOX1* | 26137 | 79618 | ENSG00000147421 | 8p21.1 |
| Candidate TSG | *HMG20A* | 5001 | 10363 | ENSG00000140382 | 15q24.3 |
| Candidate TSG | *HMG20B* | 5002 | 10362 | ENSG00000064961 | 19p13.3 |
| Candidate TSG | *HMGN5* | 8013 | 79366 | ENSG00000198157 | Xq21.1 |
| Candidate TSG | *HNRNPAB* | 5034 | 3182 | ENSG00000197451 | 5q35.3 |
| Candidate TSG | *HNRNPH1* | 5041 | 3187 | ENSG00000169045 | 5q35.3 |
| Candidate TSG | *HOMER2* | 17513 | 9455 | ENSG00000103942 | 15q25.2 |
| Candidate TSG | *HR* | 5172 | 55806 | ENSG00000168453 | 8p21.3 |
| Candidate TSG | *HS3ST5* | 19419 | 222537 | ENSG00000249853 | 6q22.1 |
| Candidate TSG | *HSD11B1L* | 30419 | 374875 | ENSG00000167733 | 19p13.3 |
| Candidate TSG | *HTR2C* | 5295 | 3358 | ENSG00000147246 | Xq23 |
| Candidate TSG | *ICMT* | 5350 | 23463 | ENSG00000116237 | 1p36.31 |
| Candidate TSG | *IDH3A* | 5384 | 3419 | ENSG00000166411 | 15q25.1 |
| Candidate TSG | *IL13RA2* | 5975 | 3598 | ENSG00000123496 | Xq23 |
| Candidate TSG | *IL16* | 5980 | 3603 | ENSG00000172349 | 15q25.1 |
| Candidate TSG | *IL17REL* | 33808 | 400935 | ENSG00000188263 | 22q13.33 |
| Candidate TSG | *IL3RA* | 6012 | 3563 | ENSG00000185291 | Xp22.33 |
| Candidate TSG | *IMP3* | 14497 | 55272 | ENSG00000177971 | 15q24.2 |
| Candidate TSG | *INPP5E* | 21474 | 56623 | ENSG00000148384 | 9q34.3 |
| Candidate TSG | *INTS10* | 25548 | 55174 | ENSG00000104613 | 8p21.3 |
| Candidate TSG | *INTS9* | 25592 | 55756 | ENSG00000104299 | 8p21.1 |
| Candidate TSG | *IQGAP2* | 6111 | 10788 | ENSG00000145703 | 5q13.3 |
| Candidate TSG | *IREB2* | 6115 | 3658 | ENSG00000136381 | 15q25.1 |
| Candidate TSG | *ISG15* | 4053 | 9636 | ENSG00000187608 | 1p36.33 |
| Candidate TSG | *ISL2* | 18524 | 64843 | ENSG00000159556 | 15q24.3 |
| Candidate TSG | *ITGB1BP3* | 17871 | 27231 | ENSG00000077009 | 19p13.3 |
| Candidate TSG | *KBTBD11* | 29104 | 9920 | ENSG00000176595 | 8p23.3 |
| Candidate TSG | *KCNAB2* | 6229 | 8514 | ENSG00000069424 | 1p36.31 |
| Candidate TSG | *KCNRG* | 18893 | 283518 | ENSG00000198553 | 13q14.2 |
| Candidate TSG | *KCTD5* | 21423 | 54442 | ENSG00000167977 | 16p13.3 |
| Candidate TSG | *KCTD9* | 22401 | 54793 | ENSG00000104756 | 8p21.2 |
| Candidate TSG | *KDM4B* | 29136 | 23030 | ENSG00000127663 | 19p13.3 |
| Candidate TSG | *KDM6A* | 12637 | 7403 | ENSG00000147050 | Xp11.3 |
| Candidate TSG | *KHSRP* | 6316 | 8570 | ENSG00000088247 | 19p13.3 |
| Candidate TSG | *KIAA0825* | 28532 | 285600 | ENSG00000185261 | 5q15 |
| Candidate TSG | *KIAA1024* | 29172 | 23251 | ENSG00000169330 | 15q25.1 |
| Candidate TSG | *KIAA1199* | 29213 | 57214 | ENSG00000103888 | 15q25.1 |
| Candidate TSG | *KIAA1751* | 29368 | 85452 | - | 1p36.33 |
| Candidate TSG | *KIAA1919* | 21053 | 91749 | ENSG00000173214 | 6q21 |
| Candidate TSG | *KIAA1967* | 23360 | 57805 | ENSG00000158941 | 8p21.3 |
| Candidate TSG | *KIAA1984* | 28236 | 84960 | ENSG00000213213 | 9q34.3 |
| Candidate TSG | *KIF13B* | 14405 | 23303 | ENSG00000197892 | 8p12 |
| Candidate TSG | *KLHDC7B* | 25145 | 113730 | ENSG00000130487 | 22q13.33 |
| Candidate TSG | *KLHL17* | 24023 | 339451 | ENSG00000187961 | 1p36.33 |
| Candidate TSG | *KLHL21* | 29041 | 9903 | ENSG00000162413 | 1p36.31 |
| Candidate TSG | *KPNA5* | 6398 | 3841 | ENSG00000196911 | 6q22.1 |
| Candidate TSG | *LACE1* | 16411 | 246269 | ENSG00000135537 | 6q21 |
| Candidate TSG | *LAMA4* | 6484 | 3910 | ENSG00000112769 | 6q21 |
| Candidate TSG | *LAMC3* | 6494 | 10319 | ENSG00000050555 | 9q34.12 |
| Candidate TSG | *LAMP1* | 6499 | 3916 | ENSG00000185896 | 13q34 |
| Candidate TSG | *LCN10* | 20892 | 414332 | ENSG00000187922 | 9q34.3 |
| Candidate TSG | *LCN12* | 28733 | 286256 | ENSG00000184925 | 9q34.3 |
| Candidate TSG | *LCN15* | 33777 | 389812 | ENSG00000177984 | 9q34.3 |
| Candidate TSG | *LCN6* | 17337 | 158062 | ENSG00000204003 | 9q34.3 |
| Candidate TSG | *LCN8* | 27038 | 138307 | ENSG00000204001 | 9q34.3 |
| Candidate TSG | *LCNL1* | 34436 | 401562 | ENSG00000214402 | 9q34.3 |
| Candidate TSG | *LGALS1* | 6561 | 3956 | ENSG00000100097 | 22q13.1 |
| Candidate TSG | *LGALS9B* | 24842 | 284194 | ENSG00000170298 | 17p11.2 |
| Candidate TSG | *LGI3* | 18711 | 203190 | ENSG00000168481 | 8p21.3 |
| Candidate TSG | *LHFP* | 6586 | 10186 | ENSG00000183722 | 13q14.11 |
| Candidate TSG | *LIN28B* | 32207 | 389421 | ENSG00000187772 | 6q16.3 |
| Candidate TSG | *LINGO1* | 21205 | 84894 | ENSG00000169783 | 15q24.3 |
| Candidate TSG | *LMAN1L* | 6632 | 79748 | ENSG00000140506 | 15q24.1 |
| Candidate TSG | *LMF2* | 25096 | 91289 | ENSG00000100258 | 22q13.33 |
| Candidate TSG | *LMNB2* | 6638 | 84823 | ENSG00000176619 | 19p13.3 |
| Candidate TSG | *LONP1* | 9479 | 9361 | ENSG00000196365 | 19p13.3 |
| Candidate TSG | *LONRF1* | 26302 | 91694 | ENSG00000154359 | 8p23.1 |
| Candidate TSG | *LOXL2* | 6666 | 4017 | ENSG00000134013 | 8p21.3 |
| Candidate TSG | *LPAR6* | 15520 | 10161 | ENSG00000139679 | 13q14.2 |
| Candidate TSG | *LPCAT4* | 30059 | 254531 | ENSG00000176454 | 15q14 |
| Candidate TSG | *LPL* | 6677 | 4023 | ENSG00000175445 | 8p21.3 |
| Candidate TSG | *LRBA* | 1742 | 987 | ENSG00000198589 | 4q31.3 |
| Candidate TSG | *LRCH2* | 29292 | 57631 | ENSG00000130224 | Xq23 |
| Candidate TSG | *LRG1* | 29480 | 116844 | ENSG00000171236 | 19p13.3 |
| Candidate TSG | *LRRC26* | 31409 | 389816 | ENSG00000184709 | 9q34.3 |
| Candidate TSG | *LUZP4* | 24971 | 51213 | ENSG00000102021 | Xq23 |
| Candidate TSG | *LZTS1* | 13861 | 11178 | ENSG00000061337 | 8p21.3 |
| Candidate TSG | *MAMDC4* | 24083 | 158056 | ENSG00000177943 | 9q34.3 |
| Candidate TSG | *MAN1A1* | 6821 | 4121 | ENSG00000111885 | 6q22.31 |
| Candidate TSG | *MAN1B1* | 6823 | 11253 | ENSG00000177239 | 9q34.3 |
| Candidate TSG | *MAN2C1* | 6827 | 4123 | ENSG00000140400 | 15q24.2 |
| Candidate TSG | *MAOA* | 6833 | 4128 | ENSG00000189221 | Xp11.3 |
| Candidate TSG | *MAOB* | 6834 | 4129 | ENSG00000069535 | Xp11.3 |
| Candidate TSG | *MAP2K2* | 6842 | 5605 | ENSG00000126934 | 19p13.3 |
| Candidate TSG | *MAP2K4* | 6844 | 6416 | ENSG00000065559 | 17p12 |
| Candidate TSG | *MAPK11* | 6873 | 5600 | ENSG00000185386 | 22q13.33 |
| Candidate TSG | *MAPK12* | 6874 | 6300 | ENSG00000188130 | 22q13.33 |
| Candidate TSG | *MAPK8IP2* | 6883 | 23542 | ENSG00000008735 | 22q13.33 |
| Candidate TSG | *MAPKBP1* | 29536 | 23005 | ENSG00000137802 | 15q15.1 |
| Candidate TSG | *MARCKS* | 6759 | 4082 | ENSG00000155130 | 6q21 |
| Candidate TSG | *MAT2B* | 6905 | 27430 | ENSG00000038274 | 5q34 |
| Candidate TSG | *MATK* | 6906 | 4145 | ENSG00000007264 | 19p13.3 |
| Candidate TSG | *MC1R* | 6929 | 4157 | ENSG00000258839 | 16q24.3 |
| Candidate TSG | *MCF2L* | 14576 | 23263 | ENSG00000126217 | 13q34 |
| Candidate TSG | *MCHR2* | 20867 | 84539 | ENSG00000152034 | 6q16.2 |
| Candidate TSG | *MCM9* | 21484 | 254394 | ENSG00000111877 | 6q22.31 |
| Candidate TSG | *MCPH1* | 6954 | 79648 | ENSG00000147316 | 8p23.1 |
| Candidate TSG | *MCTP1* | 26183 | 79772 | ENSG00000175471 | 5q15 |
| Candidate TSG | *MED9* | 25487 | 55090 | ENSG00000141026 | 17p11.2 |
| Candidate TSG | *MEF2C* | 6996 | 4208 | ENSG00000081189 | 5q14.3 |
| Candidate TSG | *MEGF6* | 3232 | 1953 | ENSG00000162591 | 1p36.32 |
| Candidate TSG | *MEIS2* | 7001 | 4212 | ENSG00000134138 | 15q14 |
| Candidate TSG | *MESDC1* | 13519 | 59274 | ENSG00000140406 | 15q25.1 |
| Candidate TSG | *MESDC2* | 13520 | 23184 | ENSG00000117899 | 15q25.1 |
| Candidate TSG | *MEX3B* | 25297 | 84206 | ENSG00000183496 | 15q25.2 |
| Candidate TSG | *MFHAS1* | 16982 | 9258 | ENSG00000147324 | 8p23.1 |
| Candidate TSG | *MGA* | 14010 | 23269 | ENSG00000174197 | 15q15.1 |
| Candidate TSG | *MICAL1* | 20619 | 64780 | ENSG00000135596 | 6q21 |
| Candidate TSG | *MIER2* | 29210 | 54531 | ENSG00000105556 | 19p13.3 |
| Candidate TSG | *MIOX* | 14522 | 55586 | ENSG00000100253 | 22q13.33 |
| Candidate TSG | *MKL1* | 14334 | 57591 | ENSG00000196588 | 22q13.2 |
| Candidate TSG | *MLC1* | 17082 | 23209 | ENSG00000100427 | 22q13.33 |
| Candidate TSG | *MLLT1* | 7134 | 4298 | ENSG00000130382 | 19p13.3 |
| Candidate TSG | *MLLT4* | 7137 | 4301 | ENSG00000130396 | 6q27 |
| Candidate TSG | *MMEL1* | 14668 | 79258 | ENSG00000142606 | 1p36.32 |
| Candidate TSG | *MORF4L1* | 16989 | 10933 | ENSG00000185787 | 15q25.1 |
| Candidate TSG | *MORN1* | 25852 | 79906 | ENSG00000116151 | 1p36.32 |
| Candidate TSG | *MOV10L1* | 7201 | 54456 | ENSG00000073146 | 22q13.33 |
| Candidate TSG | *MPI* | 7216 | 4351 | ENSG00000178802 | 15q24.1 |
| Candidate TSG | *MPND* | 25934 | 84954 | ENSG00000008382 | 19p13.3 |
| Candidate TSG | *MPRIP* | 30321 | 23164 | ENSG00000133030 | 17p11.2 |
| Candidate TSG | *MRPL41* | 14492 | 64975 | ENSG00000182154 | 9q34.3 |
| Candidate TSG | *MRPL46* | 1192 | 26589 | ENSG00000173867 | 15q25.3 |
| Candidate TSG | *MRPL54* | 16685 | 116541 | ENSG00000183617 | 19p13.3 |
| Candidate TSG | *MRPS11* | 14050 | 64963 | ENSG00000181991 | 15q25.3 |
| Candidate TSG | *MRPS31* | 16632 | 10240 | ENSG00000102738 | 13q14.11 |
| Candidate TSG | *MSH3* | 7326 | 4437 | ENSG00000113318 | 5q14.1 |
| Candidate TSG | *MSR1* | 7376 | 4481 | ENSG00000038945 | 8p22 |
| Candidate TSG | *MSRA* | 7377 | 4482 | ENSG00000175806 | 8p23.1 |
| Candidate TSG | *MTAP* | 7413 | 4507 | ENSG00000099810 | 9p21.3 |
| Candidate TSG | *MTHFD1L* | 21055 | 25902 | ENSG00000120254 | 6q25.1 |
| Candidate TSG | *MTHFS* | 7437 | 10588 | ENSG00000136371 | 15q25.1 |
| Candidate TSG | *MTMR7* | 7454 | 9108 | ENSG00000003987 | 8p22 |
| Candidate TSG | *MTMR9* | 14596 | 66036 | ENSG00000104643 | 8p23.1 |
| Candidate TSG | *MTUS1* | 29789 | 57509 | ENSG00000129422 | 8p22 |
| Candidate TSG | *MTX3* | 24812 | 345778 | ENSG00000177034 | 5q14.1 |
| Candidate TSG | *MUL1* | 25762 | 79594 | ENSG00000090432 | 1p36.12 |
| Candidate TSG | *MXRA5* | 7539 | 25878 | ENSG00000101825 | Xp22.33 |
| Candidate TSG | *MYOCD* | 16067 | 93649 | ENSG00000141052 | 17p12 |
| Candidate TSG | *MYOM2* | 7614 | 9172 | ENSG00000036448 | 8p23.3 |
| Candidate TSG | *NAT1* | 7645 | 9 | ENSG00000171428 | 8p22 |
| Candidate TSG | *NAT2* | 7646 | 10 | ENSG00000156006 | 8p22 |
| Candidate TSG | *NAT9* | 23133 | 26151 | ENSG00000109065 | 17q25.1 |
| Candidate TSG | *NBEA* | 7648 | 26960 | ENSG00000172915 | 13q13.3 |
| Candidate TSG | *NCAPH2* | 25071 | 29781 | ENSG00000025770 | 22q13.33 |
| Candidate TSG | *NCLN* | 26923 | 56926 | ENSG00000125912 | 19p13.3 |
| Candidate TSG | *NCOR1* | 7672 | 9611 | ENSG00000141027 | 17p11.2 |
| Candidate TSG | *NDFIP2* | 18537 | 54602 | ENSG00000102471 | 13q31.1 |
| Candidate TSG | *NDOR1* | 29838 | 27158 | ENSG00000188566 | 9q34.3 |
| Candidate TSG | *NDP* | 7678 | 4693 | ENSG00000124479 | Xp11.3 |
| Candidate TSG | *NDUFA11* | 20371 | 126328 | ENSG00000174886 | 19p13.3 |
| Candidate TSG | *NEFM* | 7734 | 4741 | ENSG00000104722 | 8p21.2 |
| Candidate TSG | *NEIL1* | 18448 | 79661 | ENSG00000140398 | 15q24.2 |
| Candidate TSG | *NEIL2* | 18956 | 252969 | ENSG00000154328 | 8p23.1 |
| Candidate TSG | *NELF* | 7739 | 4747 | ENSG00000104725 | 8p21.2 |
| Candidate TSG | *NELF* | 29843 | 26012 | ENSG00000165802 | 9q34.3 |
| Candidate TSG | *NF1* | 7765 | 4763 | ENSG00000196712 | 17q11.2 |
| Candidate TSG | *NFIC* | 7786 | 4782 | ENSG00000141905 | 19p13.3 |
| Candidate TSG | *NHP2* | 14377 | 55651 | ENSG00000145912 | 5q35.3 |
| Candidate TSG | *NKX2-6* | 32940 | 137814 | ENSG00000180053 | 8p21.2 |
| Candidate TSG | *NKX3-1* | 7838 | 4824 | ENSG00000167034 | 8p21.2 |
| Candidate TSG | *NLGN4X* | 14287 | 57502 | ENSG00000146938 | Xp22.31 |
| Candidate TSG | *NOC2L* | 24517 | 26155 | ENSG00000188976 | 1p36.33 |
| Candidate TSG | *NOL12* | 28585 | 79159 | ENSG00000256872 | 22q13.1 |
| Candidate TSG | *NOL9* | 26265 | 79707 | ENSG00000162408 | 1p36.31 |
| Candidate TSG | *NOP10* | 14378 | 55505 | ENSG00000182117 | 15q14 |
| Candidate TSG | *NOTCH1* | 7881 | 4851 | ENSG00000148400 | 9q34.3 |
| Candidate TSG | *NOXA1* | 10668 | 10811 | ENSG00000188747 | 9q34.3 |
| Candidate TSG | *NPDC1* | 7899 | 56654 | ENSG00000107281 | 9q34.3 |
| Candidate TSG | *NPHP4* | 19104 | 261734 | ENSG00000131697 | 1p36.31 |
| Candidate TSG | *NPM2* | 7930 | 10361 | ENSG00000158806 | 8p21.3 |
| Candidate TSG | *NPY2R* | 7957 | 4887 | ENSG00000185149 | 4q32.1 |
| Candidate TSG | *NR2E1* | 7973 | 7101 | ENSG00000112333 | 6q21 |
| Candidate TSG | *NR2F1* | 7975 | 7025 | ENSG00000175745 | 5q15 |
| Candidate TSG | *NRARP* | 33843 | 441478 | ENSG00000198435 | 9q34.3 |
| Candidate TSG | *NRG4* | 29862 | 145957 | ENSG00000169752 | 15q24.2 |
| Candidate TSG | *NRTN* | 8007 | 4902 | ENSG00000171119 | 19p13.3 |
| Candidate TSG | *NT5DC1* | 21556 | 221294 | ENSG00000178425 | 6q22.1 |
| Candidate TSG | *NT5M* | 15769 | 56953 | ENSG00000205309 | 17p11.2 |
| Candidate TSG | *NUDT18* | 26194 | 79873 | ENSG00000173566 | 8p21.3 |
| Candidate TSG | *NUP214* | 8064 | 8021 | ENSG00000126883 | 9q34.13 |
| Candidate TSG | *NUS1* | 21042 | 116150 | ENSG00000153989 | 6q22.1 |
| Candidate TSG | *ODF3B* | 34388 | 440836 | ENSG00000177989 | 22q13.33 |
| Candidate TSG | *ODF3L1* | 28735 | 161753 | ENSG00000182950 | 15q24.2 |
| Candidate TSG | *ODF3L2* | 26841 | 284451 | ENSG00000181781 | 19p13.3 |
| Candidate TSG | *OMG* | 8135 | 4974 | ENSG00000126861 | 17q11.2 |
| Candidate TSG | *OPHN1* | 8148 | 4983 | ENSG00000079482 | Xq12 |
| Candidate TSG | *OR4F16* | 15079 | 81399 | ENSG00000185097 | 1p36.33 |
| Candidate TSG | *OR4F17* | 15381 | 81099 | ENSG00000176695 | 19p13.3 |
| Candidate TSG | *OR4F21* | 19583 | 441308 | ENSG00000176269 | 8p23.3 |
| Candidate TSG | *OR4F29* | 31275 | 729759 | ENSG00000235249 | 1p36.33 |
| Candidate TSG | *OR4F3* | 8300 | 26683 | ENSG00000230178 | 5q35.3 |
| Candidate TSG | *OR4F5* | 14825 | 79501 | ENSG00000186092 | 1p36.33 |
| Candidate TSG | *OSTM1* | 21652 | 28962 | ENSG00000081087 | 6q21 |
| Candidate TSG | *P2RY8* | 15524 | 286530 | ENSG00000182162 | Xp22.33 |
| Candidate TSG | *PANK4* | 19366 | 55229 | ENSG00000157881 | 1p36.32 |
| Candidate TSG | *PANX2* | 8600 | 56666 | ENSG00000073150 | 22q13.33 |
| Candidate TSG | *PAPD4* | 26776 | 167153 | ENSG00000164329 | 5q14.1 |
| Candidate TSG | *PARK2* | 8607 | 5071 | ENSG00000185345 | 6q26 |
| Candidate TSG | *PARK7* | 16369 | 11315 | ENSG00000116288 | 1p36.23 |
| Candidate TSG | *PBK* | 18282 | 55872 | ENSG00000168078 | 8p21.1 |
| Candidate TSG | *PCID2* | 25653 | 55795 | ENSG00000126226 | 13q34 |
| Candidate TSG | *PCM1* | 8727 | 5108 | ENSG00000078674 | 8p22 |
| Candidate TSG | *PDCD2* | 8762 | 5134 | ENSG00000071994 | 6q27 |
| Candidate TSG | *PDE10A* | 8772 | 10846 | ENSG00000112541 | 6q27 |
| Candidate TSG | *PDGFRL* | 8805 | 5157 | ENSG00000104213 | 8p22 |
| Candidate TSG | *PDLIM2* | 13992 | 64236 | ENSG00000120913 | 8p21.3 |
| Candidate TSG | *PDSS2* | 23041 | 57107 | ENSG00000164494 | 6q21 |
| Candidate TSG | *PEAK1* | - | 79834 | ENSG00000173517 | 15q24.3 |
| Candidate TSG | *PEBP4* | 28319 | 157310 | ENSG00000134020 | 8p21.3 |
| Candidate TSG | *PEMT* | 8830 | 10400 | ENSG00000133027 | 17p11.2 |
| Candidate TSG | *PER3* | 8847 | 8863 | ENSG00000049246 | 1p36.23 |
| Candidate TSG | *PEX10* | 8851 | 5192 | ENSG00000157911 | 1p36.32 |
| Candidate TSG | *PGBD4* | 19401 | 161779 | ENSG00000182405 | 15q14 |
| Candidate TSG | *PHF10* | 18250 | 55274 | ENSG00000130024 | 6q27 |
| Candidate TSG | *PHF13* | 22983 | 148479 | ENSG00000116273 | 1p36.31 |
| Candidate TSG | *PHPT1* | 30033 | 29085 | ENSG00000054148 | 9q34.3 |
| Candidate TSG | *PHYHIP* | 16865 | 9796 | ENSG00000168490 | 8p21.3 |
| Candidate TSG | *PIAS4* | 17002 | 51588 | ENSG00000105229 | 19p13.3 |
| Candidate TSG | *PIGL* | 8966 | 9487 | ENSG00000108474 | 17p11.2 |
| Candidate TSG | *PIK3R1* | 8979 | 5295 | ENSG00000145675 | 5q13.1 |
| Candidate TSG | *PIM3* | 19310 | 415116 | ENSG00000198355 | 22q13.33 |
| Candidate TSG | *PINX1* | 30046 | 54984 | ENSG00000254093 | 8p23.1 |
| Candidate TSG | *PIP5K1C* | 8996 | 23396 | ENSG00000186111 | 19p13.3 |
| Candidate TSG | *PIWIL2* | 17644 | 55124 | ENSG00000197181 | 8p21.3 |
| Candidate TSG | *PJA2* | 17481 | 9867 | ENSG00000198961 | 5q21.3 |
| Candidate TSG | *PLCH2* | 29037 | 9651 | ENSG00000149527 | 1p36.32 |
| Candidate TSG | *PLD6* | 30447 | 201164 | ENSG00000179598 | 17p11.2 |
| Candidate TSG | *PLEKHG1* | 20884 | 57480 | ENSG00000120278 | 6q25.1 |
| Candidate TSG | *PLEKHG5* | 29105 | 57449 | ENSG00000171680 | 1p36.31 |
| Candidate TSG | *PLEKHN1* | 25284 | 84069 | ENSG00000187583 | 1p36.33 |
| Candidate TSG | *PLIN3* | 16893 | 10226 | ENSG00000105355 | 19p13.3 |
| Candidate TSG | *PLIN4* | 29393 | 729359 | ENSG00000167676 | 19p13.3 |
| Candidate TSG | *PLIN5* | 33196 | 440503 | ENSG00000214456 | 19p13.3 |
| Candidate TSG | *PLN* | 9080 | 5350 | ENSG00000198523 | 6q22.31 |
| Candidate TSG | *PLS3* | 9091 | 5358 | ENSG00000102024 | Xq23 |
| Candidate TSG | *PLXNB2* | 9104 | 23654 | ENSG00000196576 | 22q13.33 |
| Candidate TSG | *PMPCA* | 18667 | 23203 | ENSG00000165688 | 9q34.3 |
| Candidate TSG | *PNISR* | 21222 | 25957 | ENSG00000132424 | 6q16.2 |
| Candidate TSG | *PNMA2* | 9159 | 10687 | ENSG00000240694 | 8p21.2 |
| Candidate TSG | *PNMA3* | 18742 | 29944 | ENSG00000183837 | Xq28 |
| Candidate TSG | *PNMA6A* | 28248 | 84968 | ENSG00000224201 | Xq28 |
| Candidate TSG | *PNOC* | 9163 | 5368 | ENSG00000168081 | 8p21.1 |
| Candidate TSG | *PNPLA7* | 24768 | 375775 | ENSG00000130653 | 9q34.3 |
| Candidate TSG | *POF1B* | 13711 | 79983 | ENSG00000124429 | Xq21.2 |
| Candidate TSG | *POLR3D* | 1080 | 661 | ENSG00000168495 | 8p21.3 |
| Candidate TSG | *POPDC3* | 17649 | 64208 | ENSG00000132429 | 6q21 |
| Candidate TSG | *PPAP2C* | 9230 | 8612 | ENSG00000141934 | 19p13.3 |
| Candidate TSG | *PPCDC* | 28107 | 60490 | ENSG00000138621 | 15q24.2 |
| Candidate TSG | *PPIL6* | 21557 | 285755 | ENSG00000185250 | 6q21 |
| Candidate TSG | *PPP1R3B* | 14942 | 79660 | ENSG00000173281 | 8p23.1 |
| Candidate TSG | *PPP2CB* | 9300 | 5516 | ENSG00000104695 | 8p12 |
| Candidate TSG | *PPP2R2A* | 9304 | 5520 | ENSG00000221914 | 8p21.2 |
| Candidate TSG | *PPP2R3B* | 13417 | 28227 | ENSG00000167393 | Xp22.33 |
| Candidate TSG | *PPP3CC* | 9316 | 5533 | ENSG00000120910 | 8p21.3 |
| Candidate TSG | *PPP6R2* | 19253 | 9701 | ENSG00000100239 | 22q13.33 |
| Candidate TSG | *PRDM1* | 9346 | 639 | ENSG00000057657 | 6q21 |
| Candidate TSG | *PRDM13* | 13998 | 59336 | ENSG00000112238 | 6q16.2 |
| Candidate TSG | *PRDM16* | 14000 | 63976 | ENSG00000142611 | 1p36.32 |
| Candidate TSG | *PRDX4* | 17169 | 10549 | ENSG00000123131 | Xp22.11 |
| Candidate TSG | *PREP* | 9358 | 5550 | ENSG00000085377 | 6q21 |
| Candidate TSG | *PRKCZ* | 9412 | 5590 | ENSG00000067606 | 1p36.33 |
| Candidate TSG | *PRKX* | 9441 | 5613 | ENSG00000183943 | Xp22.33 |
| Candidate TSG | *PROZ* | 9460 | 8858 | ENSG00000126231 | 13q34 |
| Candidate TSG | *PRR22* | 28354 | 163154 | ENSG00000212123 | 19p13.3 |
| Candidate TSG | *PRR5* | 31682 | 55615 | ENSG00000186654 | 22q13.31 |
| Candidate TSG | *PRR5-ARHGAP8* | 34512 | 553158 | ENSG00000248405 | 22q13.31 |
| Candidate TSG | *PRSS27* | 15475 | 83886 | ENSG00000172382 | 16p13.3 |
| Candidate TSG | *PRSS55* | 30824 | 203074 | ENSG00000184647 | 8p23.1 |
| Candidate TSG | *PSD3* | 19093 | 23362 | ENSG00000156011 | 8p22 |
| Candidate TSG | *PSMA4* | 9533 | 5685 | ENSG00000041357 | 15q25.1 |
| Candidate TSG | *PSMB1* | 9537 | 5689 | ENSG00000008018 | 6q27 |
| Candidate TSG | *PSPN* | 9579 | 5623 | ENSG00000125650 | 19p13.3 |
| Candidate TSG | *PSTPIP1* | 9580 | 9051 | ENSG00000140368 | 15q24.3 |
| Candidate TSG | *PTEN* | 9588 | 5728 | ENSG00000171862 | 10q23.31 |
| Candidate TSG | *PTGDS* | 9592 | 5730 | ENSG00000107317 | 9q34.3 |
| Candidate TSG | *PTK2B* | 9612 | 2185 | ENSG00000120899 | 8p21.2 |
| Candidate TSG | *PTPN9* | 9661 | 5780 | ENSG00000169410 | 15q24.2 |
| Candidate TSG | *PTPRS* | 9681 | 5802 | ENSG00000105426 | 19p13.3 |
| Candidate TSG | *QRFP* | 29982 | 347148 | ENSG00000188710 | 9q34.12 |
| Candidate TSG | *QRSL1* | 21020 | 55278 | ENSG00000130348 | 6q21 |
| Candidate TSG | *RAB11FIP4* | 30267 | 84440 | ENSG00000131242 | 17q11.2 |
| Candidate TSG | *RAB37* | 30268 | 326624 | ENSG00000172794 | 17q25.1 |
| Candidate TSG | *RABL2B* | 9800 | 11158 | ENSG00000079974 | 22q13.33 |
| Candidate TSG | *RAI1* | 9834 | 10743 | ENSG00000108557 | 17p11.2 |
| Candidate TSG | *RANBP3* | 9850 | 8498 | ENSG00000031823 | 19p13.3 |
| Candidate TSG | *RASA1* | 9871 | 5921 | ENSG00000145715 | 5q14.3 |
| Candidate TSG | *RASA3* | 20331 | 22821 | ENSG00000185989 | 13q34 |
| Candidate TSG | *RASD1* | 15828 | 51655 | ENSG00000108551 | 17p11.2 |
| Candidate TSG | *RASGRF1* | 9875 | 5923 | ENSG00000058335 | 15q25.1 |
| Candidate TSG | *RASGRF2* | 9876 | 5924 | ENSG00000113319 | 5q14.1 |
| Candidate TSG | *RASGRP1* | 9878 | 10125 | ENSG00000172575 | 15q14 |
| Candidate TSG | *RAX2* | 18286 | 84839 | ENSG00000173976 | 19p13.3 |
| Candidate TSG | *RB1* | 9884 | 5925 | ENSG00000139687 | 13q14.2 |
| Candidate TSG | *RBM26* | 20327 | 64062 | ENSG00000139746 | 13q31.1 |
| Candidate TSG | *RBMXL3* | 26859 | 139804 | ENSG00000175718 | Xq23 |
| Candidate TSG | *RCBTB1* | 18243 | 55213 | ENSG00000136144 | 13q14.2 |
| Candidate TSG | *RCN2* | 9935 | 5955 | ENSG00000117906 | 15q24.3 |
| Candidate TSG | *REEP4* | 26176 | 80346 | ENSG00000168476 | 8p21.3 |
| Candidate TSG | *RER1* | 30309 | 11079 | ENSG00000157916 | 1p36.32 |
| Candidate TSG | *REV3L* | 9968 | 5980 | ENSG00000009413 | 6q21 |
| Candidate TSG | *RFPL4B* | 33264 | 442247 | ENSG00000251258 | 6q21 |
| Candidate TSG | *RFX2* | 9983 | 5990 | ENSG00000087903 | 19p13.3 |
| Candidate TSG | *RFX6* | 21478 | 222546 | ENSG00000185002 | 6q22.1 |
| Candidate TSG | *RHOBTB2* | 18756 | 23221 | ENSG00000008853 | 8p21.3 |
| Candidate TSG | *RLBP1* | 10024 | 6017 | ENSG00000140522 | 15q26.1 |
| Candidate TSG | *RMND5B* | 26181 | 64777 | ENSG00000145916 | 5q35.3 |
| Candidate TSG | *RNF207* | 32947 | 388591 | ENSG00000158286 | 1p36.31 |
| Candidate TSG | *RNF208* | 25420 | 727800 | ENSG00000212864 | 9q34.3 |
| Candidate TSG | *ROS1* | 10261 | 6098 | ENSG00000047936 | 6q22.1 |
| Candidate TSG | *RP1L1* | 15946 | 94137 | ENSG00000183638 | 8p23.1 |
| Candidate TSG | *RPF2* | 20870 | 84154 | ENSG00000197498 | 6q21 |
| Candidate TSG | *RPH3AL* | 10296 | 9501 | ENSG00000181031 | 17p13.3 |
| Candidate TSG | *RPL13* | 10303 | 6137 | ENSG00000167526 | 16q24.3 |
| Candidate TSG | *RPL22* | 10315 | 6146 | ENSG00000116251 | 1p36.31 |
| Candidate TSG | *RPL23AP82* | 33730 | 284942 | - | 22q13.33 |
| Candidate TSG | *RPL36* | 13631 | 25873 | ENSG00000130255 | 19p13.3 |
| Candidate TSG | *RPP25* | 30361 | 54913 | ENSG00000178718 | 15q24.2 |
| Candidate TSG | *RPS17* | 10397 | 6218 | ENSG00000184779 | 15q25.2 |
| Candidate TSG | *RPS23* | 10410 | 6228 | ENSG00000186468 | 5q14.2 |
| Candidate TSG | *RPS6KA6* | 10435 | 27330 | ENSG00000072133 | Xq21.1 |
| Candidate TSG | *RSPH4A* | 21558 | 345895 | ENSG00000111834 | 6q22.1 |
| Candidate TSG | *RTN4IP1* | 18647 | 84816 | ENSG00000130347 | 6q21 |
| Candidate TSG | *RTTN* | 18654 | 25914 | ENSG00000176225 | 18q22.2 |
| Candidate TSG | *RUFY1* | 19760 | 80230 | ENSG00000176783 | 5q35.3 |
| Candidate TSG | *RWDD1* | 20993 | 51389 | ENSG00000111832 | 6q22.1 |
| Candidate TSG | *RYR3* | 10485 | 6263 | ENSG00000198838 | 15q14 |
| Candidate TSG | *S100Z* | 30367 | 170591 | ENSG00000171643 | 5q13.3 |
| Candidate TSG | *S1PR4* | 3170 | 8698 | ENSG00000125910 | 19p13.3 |
| Candidate TSG | *SAFB* | 10520 | 6294 | ENSG00000160633 | 19p13.3 |
| Candidate TSG | *SAFB2* | 21605 | 9667 | ENSG00000130254 | 19p13.3 |
| Candidate TSG | *SAMD11* | 28706 | 148398 | ENSG00000187634 | 1p36.33 |
| Candidate TSG | *SASH1* | 19182 | 23328 | ENSG00000111961 | 6q24.3 |
| Candidate TSG | *SAT1* | 10540 | 6303 | ENSG00000130066 | Xp22.11 |
| Candidate TSG | *SATL1* | 27992 | 340562 | ENSG00000184788 | Xq21.1 |
| Candidate TSG | *SBF1* | 10542 | 6305 | ENSG00000100241 | 22q13.33 |
| Candidate TSG | *SCAMP2* | 10564 | 10066 | ENSG00000140497 | 15q24.1 |
| Candidate TSG | *SCAMP5* | 30386 | 192683 | ENSG00000198794 | 15q24.2 |
| Candidate TSG | *SCAPER* | 13081 | 49855 | ENSG00000140386 | 15q24.3 |
| Candidate TSG | *SCARA3* | 19000 | 51435 | ENSG00000168077 | 8p21.1 |
| Candidate TSG | *SCARA5* | 28701 | 286133 | ENSG00000168079 | 8p21.1 |
| Candidate TSG | *SCML4* | 21397 | 256380 | ENSG00000146285 | 6q21 |
| Candidate TSG | *SCO2* | 10604 | 9997 | ENSG00000130489 | 22q13.33 |
| Candidate TSG | *SDCCAG3* | 10667 | 10807 | ENSG00000165689 | 9q34.3 |
| Candidate TSG | *SEC63* | 21082 | 11231 | ENSG00000025796 | 6q21 |
| Candidate TSG | *SELO* | - | 83642 | ENSG00000073169 | 22q13.33 |
| Candidate TSG | *SEMA6B* | 10739 | 10501 | ENSG00000167680 | 19p13.3 |
| Candidate TSG | *SERINC5* | 18825 | 256987 | ENSG00000164300 | 5q14.1 |
| Candidate TSG | *SESN1* | 21595 | 27244 | ENSG00000080546 | 6q21 |
| Candidate TSG | *SFTPC* | 10802 | 6440 | ENSG00000168484 | 8p21.3 |
| Candidate TSG | *SGCZ* | 14075 | 137868 | ENSG00000185053 | 8p22 |
| Candidate TSG | *SGK223* | - | 157285 | ENSG00000182319 | 8p23.1 |
| Candidate TSG | *SGTA* | 10819 | 6449 | ENSG00000104969 | 19p13.3 |
| Candidate TSG | *SH2D3A* | 16885 | 10045 | ENSG00000125731 | 19p13.3 |
| Candidate TSG | *SH2D4A* | 26102 | 63898 | ENSG00000104611 | 8p21.3 |
| Candidate TSG | *SH2D7* | 34549 | 646892 | ENSG00000183476 | 15q25.1 |
| Candidate TSG | *SH3BGRL* | 10823 | 6451 | ENSG00000131171 | Xq21.1 |
| Candidate TSG | *SH3GL1* | 10830 | 6455 | ENSG00000141985 | 19p13.3 |
| Candidate TSG | *SH3GL3* | 10832 | 6457 | ENSG00000140600 | 15q25.2 |
| Candidate TSG | *SHANK3* | 14294 | 85358 | ENSG00000251322 | 22q13.33 |
| Candidate TSG | *SHC2* | 29869 | 25759 | ENSG00000129946 | 19p13.3 |
| Candidate TSG | *SHD* | 30633 | 56961 | ENSG00000105251 | 19p13.3 |
| Candidate TSG | *SHISA6* | 34491 | 388336 | ENSG00000188803 | 17p12 |
| Candidate TSG | *SHOX* | 10853 | 6473 | ENSG00000185960 | Xp22.33 |
| Candidate TSG | *SIM1* | 10882 | 6492 | ENSG00000112246 | 6q16.3 |
| Candidate TSG | *SIN3A* | 19353 | 25942 | ENSG00000169375 | 15q24.2 |
| Candidate TSG | *SIRT6* | 14934 | 51548 | ENSG00000077463 | 19p13.3 |
| Candidate TSG | *SKI* | 10896 | 6497 | ENSG00000157933 | 1p36.33 |
| Candidate TSG | *SLC12A6* | 10914 | 9990 | ENSG00000140199 | 15q14 |
| Candidate TSG | *SLC16A10* | 17027 | 117247 | ENSG00000112394 | 6q21 |
| Candidate TSG | *SLC18A1* | 10934 | 6570 | ENSG00000036565 | 8p21.3 |
| Candidate TSG | *SLC22A16* | 20302 | 85413 | ENSG00000004809 | 6q21 |
| Candidate TSG | *SLC25A15* | 10985 | 10166 | ENSG00000102743 | 13q14.11 |
| Candidate TSG | *SLC25A23* | 19375 | 79085 | ENSG00000125648 | 19p13.3 |
| Candidate TSG | *SLC25A37* | 29786 | 51312 | ENSG00000147454 | 8p21.2 |
| Candidate TSG | *SLC25A41* | 28533 | 284427 | ENSG00000181240 | 19p13.3 |
| Candidate TSG | *SLC25A6* | 10992 | 293 | ENSG00000169100 | Xp22.33 |
| Candidate TSG | *SLC34A3* | 20305 | 142680 | ENSG00000198569 | 9q34.3 |
| Candidate TSG | *SLC35F1* | 21483 | 222553 | ENSG00000196376 | 6q22.1 |
| Candidate TSG | *SLC35G5* | 15546 | 83650 | ENSG00000177710 | 8p23.1 |
| Candidate TSG | *SLC39A14* | 20858 | 23516 | ENSG00000104635 | 8p21.3 |
| Candidate TSG | *SLC39A3* | 17128 | 29985 | ENSG00000141873 | 19p13.3 |
| Candidate TSG | *SLC47A1* | 25588 | 55244 | ENSG00000142494 | 17p11.2 |
| Candidate TSG | *SLC47A2* | 26439 | 146802 | ENSG00000180638 | 17p11.2 |
| Candidate TSG | *SLC7A2* | 11060 | 6542 | ENSG00000003989 | 8p22 |
| Candidate TSG | *SLC9A3R1* | 11075 | 9368 | ENSG00000109062 | 17q25.1 |
| Candidate TSG | *SMAD9* | 6774 | 4093 | ENSG00000120693 | 13q13.3 |
| Candidate TSG | *SMOC2* | 20323 | 64094 | ENSG00000112562 | 6q27 |
| Candidate TSG | *SMPD2* | 11121 | 6610 | ENSG00000135587 | 6q21 |
| Candidate TSG | *SNAPC4* | 11137 | 6621 | ENSG00000165684 | 9q34.3 |
| Candidate TSG | *SNUPN* | 14245 | 10073 | ENSG00000169371 | 15q24.2 |
| Candidate TSG | *SNX3* | 11174 | 8724 | ENSG00000112335 | 6q21 |
| Candidate TSG | *SNX33* | 28468 | 257364 | ENSG00000173548 | 15q24.2 |
| Candidate TSG | *SOBP* | 29256 | 55084 | ENSG00000112320 | 6q21 |
| Candidate TSG | *SOCS6* | 16833 | 9306 | ENSG00000170677 | 18q22.2 |
| Candidate TSG | *SORBS3* | 30907 | 10174 | ENSG00000120896 | 8p21.3 |
| Candidate TSG | *SOX1* | 11189 | 6656 | ENSG00000182968 | 13q34 |
| Candidate TSG | *SOX7* | 18196 | 83595 | ENSG00000171056 | 8p23.1 |
| Candidate TSG | *SPACA7* | 29575 | 122258 | ENSG00000153498 | 13q34 |
| Candidate TSG | *SPAG11A* | 33342 | 653423 | ENSG00000178287 | 8p23.1 |
| Candidate TSG | *SPAG11B* | 14534 | 10407 | ENSG00000164871 | 8p23.1 |
| Candidate TSG | *SPATA2L* | 28393 | 124044 | ENSG00000158792 | 16q24.3 |
| Candidate TSG | *SPECC1* | 30615 | 92521 | ENSG00000128487 | 17p11.2 |
| Candidate TSG | *SPG7* | 11237 | 6687 | ENSG00000197912 | 16q24.3 |
| Candidate TSG | *SPIRE2* | 30623 | 84501 | ENSG00000204991 | 16q24.3 |
| Candidate TSG | *SPPL2B* | - | 56928 | ENSG00000005206 | 19p13.3 |
| Candidate TSG | *SPRED1* | 20249 | 161742 | ENSG00000166068 | 15q14 |
| Candidate TSG | *SPRY2* | 11270 | 10253 | ENSG00000136158 | 13q31.1 |
| Candidate TSG | *SPRYD7* | 14297 | 57213 | ENSG00000123178 | 13q14.2 |
| Candidate TSG | *SPZ1* | 30721 | 84654 | ENSG00000164299 | 5q14.1 |
| Candidate TSG | *SREBF1* | 11289 | 6720 | ENSG00000072310 | 17p11.2 |
| Candidate TSG | *SSBP2* | 15831 | 23635 | ENSG00000145687 | 5q14.1 |
| Candidate TSG | *SSNA1* | 11321 | 8636 | ENSG00000176101 | 9q34.3 |
| Candidate TSG | *ST20* | 33520 | 400410 | ENSG00000180953 | 15q25.1 |
| Candidate TSG | *STAG2* | 11355 | 10735 | ENSG00000101972 | Xq25 |
| Candidate TSG | *STAP2* | 30430 | 55620 | ENSG00000178078 | 19p13.3 |
| Candidate TSG | *STARD5* | 18065 | 80765 | ENSG00000172345 | 15q25.1 |
| Candidate TSG | *STC1* | 11373 | 6781 | ENSG00000159167 | 8p21.2 |
| Candidate TSG | *STMN4* | 16078 | 81551 | ENSG00000015592 | 8p21.2 |
| Candidate TSG | *SYNE1* | 17089 | 23345 | ENSG00000131018 | 6q25.2 |
| Candidate TSG | *T* | 11515 | 6862 | ENSG00000164458 | 6q27 |
| Candidate TSG | *TAS1R1* | 14448 | 80835 | ENSG00000173662 | 1p36.31 |
| Candidate TSG | *TBC1D2B* | 29183 | 23102 | ENSG00000167202 | 15q25.1 |
| Candidate TSG | *TBP* | 11588 | 6908 | ENSG00000112592 | 6q27 |
| Candidate TSG | *TBXA2R* | 11608 | 6915 | ENSG00000006638 | 19p13.3 |
| Candidate TSG | *TCF25* | 29181 | 22980 | ENSG00000141002 | 16q24.3 |
| Candidate TSG | *TCTE3* | 11695 | 6991 | ENSG00000184786 | 6q27 |
| Candidate TSG | *TEX13B* | 11736 | 56156 | ENSG00000170925 | Xq22.3 |
| Candidate TSG | *TEX15* | 11738 | 56154 | ENSG00000133863 | 8p12 |
| Candidate TSG | *TFDP1* | 11749 | 7027 | ENSG00000198176 | 13q34 |
| Candidate TSG | *THAP3* | 20855 | 90326 | ENSG00000041988 | 1p36.31 |
| Candidate TSG | *THBS2* | 11786 | 7058 | ENSG00000186340 | 6q27 |
| Candidate TSG | *THBS4* | 11788 | 7060 | ENSG00000113296 | 5q14.1 |
| Candidate TSG | *THEG* | 13706 | 51298 | ENSG00000105549 | 19p13.3 |
| Candidate TSG | *THOP1* | 11793 | 7064 | ENSG00000172009 | 19p13.3 |
| Candidate TSG | *THRA* | 11796 | 7067 | ENSG00000126351 | 17q21.1 |
| Candidate TSG | *TICAM1* | 18348 | 148022 | ENSG00000127666 | 19p13.3 |
| Candidate TSG | *TIMM13* | 11816 | 26517 | ENSG00000099800 | 19p13.3 |
| Candidate TSG | *TJP3* | 11829 | 27134 | ENSG00000105289 | 19p13.3 |
| Candidate TSG | *TLE6* | 30788 | 79816 | ENSG00000104953 | 19p13.3 |
| Candidate TSG | *TM6SF1* | 11860 | 53346 | ENSG00000136404 | 15q25.2 |
| Candidate TSG | *TMC3* | 22995 | 342125 | ENSG00000188869 | 15q25.1 |
| Candidate TSG | *TMCO3* | 20329 | 55002 | ENSG00000150403 | 13q34 |
| Candidate TSG | *TMCO5A* | 28558 | 145942 | ENSG00000166069 | 15q14 |
| Candidate TSG | *TMED3* | 28889 | 23423 | ENSG00000166557 | 15q25.1 |
| Candidate TSG | *TMEM104* | 25984 | 54868 | ENSG00000109066 | 17q25.1 |
| Candidate TSG | *TMEM141* | 28211 | 85014 | ENSG00000244187 | 9q34.3 |
| Candidate TSG | *TMEM146* | 28598 | 257062 | ENSG00000174898 | 19p13.3 |
| Candidate TSG | *TMEM161B* | 28483 | 153396 | ENSG00000164180 | 5q14.3 |
| Candidate TSG | *TMEM167A* | 28330 | 153339 | ENSG00000174695 | 5q14.2 |
| Candidate TSG | *TMEM203* | 28217 | 94107 | ENSG00000187713 | 9q34.3 |
| Candidate TSG | *TMEM52* | 27916 | 339456 | ENSG00000178821 | 1p36.33 |
| Candidate TSG | *TMEM85* | 28032 | 51234 | ENSG00000128463 | 15q14 |
| Candidate TSG | *TMIGD2* | 28324 | 126259 | ENSG00000167664 | 19p13.3 |
| Candidate TSG | *TMPRSS9* | 30079 | 360200 | ENSG00000178297 | 19p13.3 |
| Candidate TSG | *TNFAIP8L1* | 28279 | 126282 | ENSG00000185361 | 19p13.3 |
| Candidate TSG | *TNFRSF10A* | 11904 | 8797 | ENSG00000104689 | 8p21.3 |
| Candidate TSG | *TNFRSF10B* | 11905 | 8795 | ENSG00000120889 | 8p21.3 |
| Candidate TSG | *TNFRSF10C* | 11906 | 8794 | ENSG00000173535 | 8p21.3 |
| Candidate TSG | *TNFRSF10D* | 11907 | 8793 | ENSG00000173530 | 8p21.3 |
| Candidate TSG | *TNFRSF13B* | 18153 | 23495 | ENSG00000240505 | 17p11.2 |
| Candidate TSG | *TNFRSF14* | 11912 | 8764 | ENSG00000157873 | 1p36.32 |
| Candidate TSG | *TNFRSF25* | 11910 | 8718 | ENSG00000215788 | 1p36.31 |
| Candidate TSG | *TNFRSF9* | 11924 | 3604 | ENSG00000049249 | 1p36.23 |
| Candidate TSG | *TNFSF14* | 11930 | 8740 | ENSG00000125735 | 19p13.3 |
| Candidate TSG | *TNFSF9* | 11939 | 8744 | ENSG00000125657 | 19p13.3 |
| Candidate TSG | *TNKS* | 11941 | 8658 | ENSG00000173273 | 8p23.1 |
| Candidate TSG | *TOM1L2* | 11984 | 146691 | ENSG00000175662 | 17p11.2 |
| Candidate TSG | *TPRN* | 26894 | 286262 | ENSG00000176058 | 9q34.3 |
| Candidate TSG | *TRABD* | 28805 | 80305 | ENSG00000170638 | 22q13.33 |
| Candidate TSG | *TRAF2* | 12032 | 7186 | ENSG00000127191 | 9q34.3 |
| Candidate TSG | *TRAF3IP2* | 1343 | 10758 | ENSG00000056972 | 6q21 |
| Candidate TSG | *TRIM13* | 9976 | 10206 | ENSG00000204977 | 13q14.2 |
| Candidate TSG | *TRIM35* | 16285 | 23087 | ENSG00000104228 | 8p21.2 |
| Candidate TSG | *TRIML1* | 26698 | 339976 | ENSG00000184108 | 4q35.2 |
| Candidate TSG | *TRIML2* | 26378 | 205860 | ENSG00000179046 | 4q35.2 |
| Candidate TSG | *TRIOBP* | 17009 | 11078 | ENSG00000100106 | 22q13.1 |
| Candidate TSG | *TRIP10* | 12304 | 9322 | ENSG00000125733 | 19p13.3 |
| Candidate TSG | *TRPV2* | 18082 | 51393 | ENSG00000187688 | 17p11.2 |
| Candidate TSG | *TSPAN3* | 17752 | 10099 | ENSG00000140391 | 15q24.3 |
| Candidate TSG | *TSPYL1* | 12382 | 7259 | ENSG00000189241 | 6q22.1 |
| Candidate TSG | *TTC37* | 23639 | 9652 | ENSG00000198677 | 5q15 |
| Candidate TSG | *TTLL8* | 34000 | 164714 | ENSG00000138892 | 22q13.33 |
| Candidate TSG | *TUBB2C* | 20771 | 10383 | ENSG00000188229 | 9q34.3 |
| Candidate TSG | *TUBB3* | 20772 | 10381 | ENSG00000198211 | 16q24.3 |
| Candidate TSG | *TUBB4* | 20774 | 10382 | ENSG00000104833 | 19p13.3 |
| Candidate TSG | *TUBB4Q* | 12413 | 56604 | ENSG00000127589 | 8p12 |
| Candidate TSG | *TUBE1* | 20775 | 51175 | ENSG00000074935 | 6q21 |
| Candidate TSG | *TUBGCP3* | 18598 | 10426 | ENSG00000126216 | 13q34 |
| Candidate TSG | *TUBGCP6* | 18127 | 85378 | ENSG00000128159 | 22q13.33 |
| Candidate TSG | *TUSC3* | 30242 | 7991 | ENSG00000104723 | 8p22 |
| Candidate TSG | *TYMP* | 3148 | 1890 | ENSG00000025708 | 22q13.33 |
| Candidate TSG | *UAP1L1* | 28082 | 91373 | ENSG00000197355 | 9q34.3 |
| Candidate TSG | *UBB* | 12463 | 7314 | ENSG00000170315 | 17p11.2 |
| Candidate TSG | *UBE2Q2* | 19248 | 92912 | ENSG00000140367 | 15q24.2 |
| Candidate TSG | *UBXN10* | 26354 | 127733 | ENSG00000162543 | 1p36.12 |
| Candidate TSG | *UBXN6* | 14928 | 80700 | ENSG00000167671 | 19p13.3 |
| Candidate TSG | *UBXN8* | 30307 | 7993 | ENSG00000104691 | 8p12 |
| Candidate TSG | *UHRF1* | 12556 | 29128 | ENSG00000034063 | 19p13.3 |
| Candidate TSG | *ULK2* | 13480 | 9706 | ENSG00000083290 | 17p11.2 |
| Candidate TSG | *ULK3* | 19703 | 25989 | ENSG00000140474 | 15q24.1 |
| Candidate TSG | *UPF3A* | 20332 | 65110 | ENSG00000169062 | 13q34 |
| Candidate TSG | *USP17L2* | 34434 | 377630 | ENSG00000223443 | 8p23.1 |
| Candidate TSG | *USP22* | 12621 | 23326 | ENSG00000124422 | 17p11.2 |
| Candidate TSG | *USP45* | 20080 | 85015 | ENSG00000123552 | 6q16.2 |
| Candidate TSG | *USP9X* | 12632 | 8239 | ENSG00000124486 | Xp11.4 |
| Candidate TSG | *UTS2* | 12636 | 10911 | ENSG00000049247 | 1p36.23 |
| Candidate TSG | *VAMP3* | 12644 | 9341 | ENSG00000049245 | 1p36.23 |
| Candidate TSG | *VAV1* | 12657 | 7409 | ENSG00000141968 | 19p13.3 |
| Candidate TSG | *VCAN* | 2464 | 1462 | ENSG00000038427 | 5q14.2 |
| Candidate TSG | *VGLL2* | 20232 | 245806 | ENSG00000170162 | 6q22.1 |
| Candidate TSG | *VMAC* | 33803 | 400673 | ENSG00000187650 | 19p13.3 |
| Candidate TSG | *VPS37A* | 24928 | 137492 | ENSG00000155975 | 8p22 |
| Candidate TSG | *VSIG4* | 17032 | 11326 | ENSG00000155659 | Xq12 |
| Candidate TSG | *VWA5B1* | 26538 | 127731 | ENSG00000158816 | 1p36.12 |
| Candidate TSG | *WASF1* | 12732 | 8936 | ENSG00000112290 | 6q21 |
| Candidate TSG | *WDR27* | 21248 | 253769 | ENSG00000184465 | 6q27 |
| Candidate TSG | *WDR61* | 30300 | 80349 | ENSG00000140395 | 15q25.1 |
| Candidate TSG | *WDR85* | 25199 | 92715 | ENSG00000148399 | 9q34.3 |
| Candidate TSG | *WHAMM* | 30493 | 123720 | ENSG00000156232 | 15q25.2 |
| Candidate TSG | *WISP3* | 12771 | 8838 | ENSG00000112761 | 6q21 |
| Candidate TSG | *WWC1* | 29435 | 23286 | ENSG00000113645 | 5q34 |
| Candidate TSG | *XKR5* | 20782 | 389610 | ENSG00000186530 | 8p23.1 |
| Candidate TSG | *XKR6* | 27806 | 286046 | ENSG00000171044 | 8p23.1 |
| Candidate TSG | *XPO7* | 14108 | 23039 | ENSG00000130227 | 8p21.3 |
| Candidate TSG | *XRCC4* | 12831 | 7518 | ENSG00000152422 | 5q14.2 |
| Candidate TSG | *YIPF6* | 28304 | 286451 | ENSG00000181704 | Xq12 |
| Candidate TSG | *ZBED4* | 20721 | 9889 | ENSG00000100426 | 22q13.33 |
| Candidate TSG | *ZBTB24* | 21143 | 9841 | ENSG00000112365 | 6q21 |
| Candidate TSG | *ZBTB48* | 4930 | 3104 | ENSG00000204859 | 1p36.31 |
| Candidate TSG | *ZBTB7A* | 18078 | 51341 | ENSG00000178951 | 19p13.3 |
| Candidate TSG | *ZCCHC9* | 25424 | 84240 | ENSG00000131732 | 5q14.1 |
| Candidate TSG | *ZDHHC2* | 18469 | 51201 | ENSG00000104219 | 8p22 |
| Candidate TSG | *ZFAND6* | 30164 | 54469 | ENSG00000086666 | 15q25.1 |
| Candidate TSG | *ZFP42* | 30949 | 132625 | ENSG00000179059 | 4q35.2 |
| Candidate TSG | *ZFR2* | 29189 | 23217 | ENSG00000105278 | 19p13.3 |
| Candidate TSG | *ZFYVE16* | 20756 | 9765 | ENSG00000039319 | 5q14.1 |
| Candidate TSG | *ZMYND19* | 21146 | 116225 | ENSG00000165724 | 9q34.3 |
| Candidate TSG | *ZNF18* | 12969 | 7566 | ENSG00000154957 | 17p12 |
| Candidate TSG | *ZNF276* | 23330 | 92822 | ENSG00000158805 | 16q24.3 |
| Candidate TSG | *ZNF287* | 13502 | 57336 | ENSG00000141040 | 17p11.2 |
| Candidate TSG | *ZNF395* | 18737 | 55893 | ENSG00000186918 | 8p21.1 |
| Candidate TSG | *ZNF554* | 26629 | 115196 | ENSG00000172006 | 19p13.3 |
| Candidate TSG | *ZNF555* | 28382 | 148254 | ENSG00000186300 | 19p13.3 |
| Candidate TSG | *ZNF556* | 25669 | 80032 | ENSG00000172000 | 19p13.3 |
| Candidate TSG | *ZNF57* | 13125 | 126295 | ENSG00000171970 | 19p13.3 |
| Candidate TSG | *ZNF596* | 27268 | 169270 | ENSG00000172748 | 8p23.3 |
| Candidate TSG | *ZNF624* | 29254 | 57547 | ENSG00000197566 | 17p11.2 |
| Candidate TSG | *ZNF705D* | 33202 | 728957 | ENSG00000215343 | 8p23.1 |
| Candidate TSG | *ZNF711* | 13128 | 7552 | ENSG00000147180 | Xq21.1 |
| Candidate TSG | *ZNF77* | 13150 | 58492 | ENSG00000175691 | 19p13.3 |
| Candidate TSG | *ZNF770* | 26061 | 54989 | ENSG00000198146 | 15q14 |
| Candidate TSG | *ZNF778* | 26479 | 197320 | ENSG00000170100 | 16q24.3 |
| Candidate TSG | *ZNF828* | 20311 | 283489 | ENSG00000198824 | 13q34 |
| Candidate TSG | *ZNRF3* | 18126 | 84133 | ENSG00000183579 | 22q12.1 |
| Candidate TSG | *ZNRF4* | 17726 | 148066 | ENSG00000105428 | 19p13.3 |
| Candidate TSG | *ZUFSP* | 21224 | 221302 | ENSG00000153975 | 6q22.1 |
